# Dax1 and Nanog act in parallel to stabilize mouse embryonic stem cells and induced pluripotency

**DOI:** 10.1038/ncomms6042

**Published:** 2014-10-06

**Authors:** Junlei Zhang, Gaoke Liu, Yan Ruan, Jiali Wang, Ke Zhao, Ying Wan, Bing Liu, Hongting Zheng, Tao Peng, Wei Wu, Ping He, Fu-Quan Hu, Rui Jian

**Affiliations:** 1 Department of Microbiology, Third Military Medical University, Chongqing 400038, China; 2 Department of Pathogenic Biology, Third Military Medical University, Chongqing 400038, China; 3 Department of Physiology, Third Military Medical University, Chongqing 400038, China; 4 Laboratory of Oncology, Affiliated Hospital of Academy of Military Medical Sciences, Beijing 400038, China; 5 Biomedical Analysis Center, Third Military Medical University, Chongqing 100071, China; 6 Department of Immunology, Third Military Medical University, Chongqing 400038, China; 7 Department of Endocrinology, Xinqiao Hospital, Third Military Medical University, Chongqing 400037, China; 8 Research Center of Laboratory Medicine, Chengdu Military General Hospital, Sichuan 610083, China; 9 Department of Cardiothoracic Surgery, Southwest Hospital, Third Military Medical University, Chongqing 400038, China

## Abstract

Nanog expression is heterogeneous and dynamic in embryonic stem cells (ESCs). However, the mechanism for stabilizing pluripotency during the transitions between Nanog^high^ and Nanog^low^ states is not well understood. Here we report that Dax1 acts in parallel with Nanog to regulate mouse ESC (mESCs) identity. Dax1 stable knockdown mESCs are predisposed towards differentiation but do not lose pluripotency, whereas Dax1 overexpression supports LIF-independent self-renewal. Although partially complementary, Dax1 and Nanog function independently and cannot replace one another. They are both required for full reprogramming to induce pluripotency. Importantly, Dax1 is indispensable for self-renewal of Nanog^low^ mESCs. Moreover, we report that Dax1 prevents extra-embryonic endoderm (ExEn) commitment by directly repressing *Gata6* transcription. Dax1 may also mediate inhibition of trophectoderm differentiation independent or as a downstream effector of Oct4. These findings establish a basal role of Dax1 in maintaining pluripotency during the state transition of mESCs and somatic cell reprogramming.

## Abstract (web summary)

The transcription factor Dax1 is often used as an embryonic stem cell (ESC) marker. Here the authors show that Dax1 acts in parallel to the pluripotency transcription factor Nanog in the maintenance of mouse ESC pluripotency and is required for full somatic cell reprogramming.

## Graphical abstract

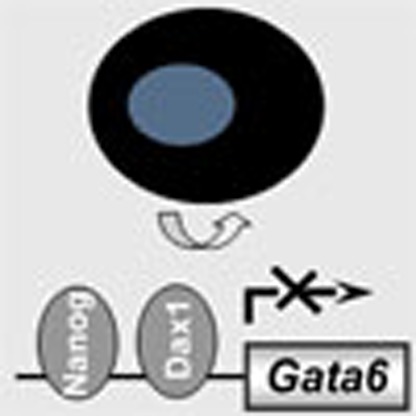

Embryonic stem cells (ESCs), derived from the inner cell mass of blastocyst-stage embryos, can maintain self-renewal and multilineage differentiation potential *in vitro*^1^. Understanding mechanisms for maintenance of pluripotency not only promotes advances in tissue engineering, embryonic development and cancer research^2^, but also allows generation of induced pluripotent stem cell (iPSC) technology^3^. Oct4 and Nanog are the most well-characterized pluripotency factors in ESCs. The Oct4/Nanog-centred regulatory network has been shown to play key roles in ESC fate determination^4^,^5^. However, while the list of network factors is rapidly expanding^6^,^7^, the functional relationships of these factors with Oct4 and Nanog are largely unknown.

Overexpression (OE) of Nanog blocks the differentiation of ESCs into extra-embryonic endoderm (ExEn), and confers cytokine-independent self-renewal^8^,^9^. Mutual antagonism between Gata6 and Nanog is thought to determine ExEn versus stem cell fate^10^. In particular, excess Gata6 gives rise to the ExEn lineage^11^, whereas excess Nanog results in the stem cell state. Unlike Nanog, Oct4 expression must be tightly regulated to maintain ESC pluripotency. Reduction of Oct4 expression below 50% induces differentiation into trophectoderm (TE) by upregulating transcription factors *Cdx2* and *Eomes*. When Oct4 expression exceeds 150%, ESCs differentiate chiefly into ExEn cells with increased Gata6 expression^12^,^13^.

Compared with the relatively uniform expression of Oct4, Nanog expression is not only heterogeneous but also dynamic in ESCs^14^,^15^. Nanog^high^ ESCs possess high self-renewal efficiency, whereas Nanog^low^ ESCs have increased propensity to ExEn differentiation and upregulated Gata6, but do not undergo commitment^15^. So far, little is known about the mechanism for stabilizing pluripotency during the transition between Nanog^high^ and Nanog^low^ states. Nanog^low^ or even Nanog^null^ ESCs retain a self-renewal capacity and pluripotency, suggesting that increased Gata6 is insufficient to induce ExEn differentiation. Therefore, possibly other ESC-specific transcription factors, which should be functionally independent of Nanog, may be involved in inhibiting full activation of Gata6 and ExEn differentiation in Nanog^low/null^ ESCs.

Dax1 (dosage-sensitive sex reversal-AHC critical region on the X-chromosome gene 1, also known as *Nr0b1*) is a transcriptional repressor^16^ that has been identified as a major component in both Oct4-centred and Nanog-centred protein interaction networks^17^,^18^,^19^. However, its mechanisms of action with respect to pluripotency are not well understood (Supplementary Table 1). Here, through systematic molecular and cellular functional analysis, we uncover a previously unknown functional relationship between Dax1 and Nanog, and suggest that these two factors function synergistically to stabilize both ESC and induced pluripotency.

## Results

### Dax1-knockdown ESCs can maintain self-renewal

**Figure 1:**
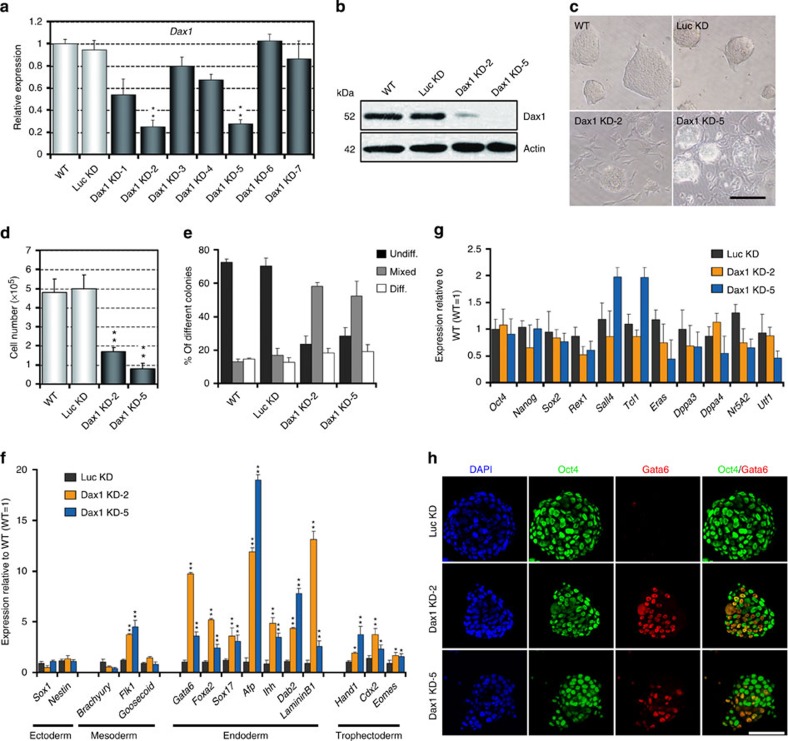
Dax1 knockdown predisposes ESCs to differentiation but with no loss of self-renewal capacity. (**a**,**b**) qRT–PCR (**a**) and immunoblot (**b**) analyses after Dax1 KD. (**c**) Morphology of colonies formed by the indicated lines. Cells were grown with LIF for three passages after zeocin selection. Scale bar, 100 μm. (**d**) ESCs (200 cells per cm^2^ in 12-well plates) were cultured for 5 days with LIF and cell numbers were counted. (**e**) Quantitative analysis of colony formation assay in the indicated lines. Cells were plated at clonal density and cultured for 6 days with LIF. Colonies were fixed and stained for AP and scored as undifferentiated (undiff.), mixed or differentiated (diff.). (**f**,**g**) qRT–PCR analyses of germ layer (**f**) and pluripotency (**g**) marker expression levels in the indicated lines cultured with LIF. All data are normalized to *Gapdh* and shown relative to WT ESCs (set at 1.0). (**h**) Immunofluorescence analysis of Oct4 (green) and Gata6 (red) in the indicated lines. Cells were cultured with LIF for 5 days and counterstained with DAPI (blue). Scale bar, 100 μm. Data in **a**,**d**–**g** are represented as mean±s.d.; *n*=3. *P<0.05; **P<0.01. All *P* values were calculated using Student’s *t*-test.

The *Dax1* gene was expressed highly in ESCs and was rapidly downregulated during differentiation^20^,^21^, suggesting a functional role for Dax1 in maintaining pluripotency of ESCs. To investigate this, we used short hairpin RNA (shRNA) lentiviral vectors to stably knockdown (KD) Dax1 in ESCs. Seven shRNAs targeting different regions of *Dax1* complementary DNA were tested. Dax1 was effectively silenced by two constructs, designated Dax1 KD-2 and Dax1 KD-5, which target the CDS (coding sequence) and 3′ untranslated region (3′ UTR), respectively (Fig. 1a,b; Supplementary Fig. 1a,b). We found that the depletion of Dax1 resulted in more differentiation-like cells and slower cell expansion compared with wild-type (WT) and luciferase KD (Luc KD, serving as a negative control) cells (Fig. 1c,d). However, Dax1 KD cells could be continuously propagated (for at least 30 passages) in the presence of leukaemia inhibitory factor (LIF) and retained the capacity to form ESC colonies (Fig. 1c). Colony formation assays showed that Dax1 KD cells formed less wholly undifferentiated alkaline phosphatase (AP)-positive colonies and more mixed (partially differentiated) colonies compared with control cells (Fig. 1e; Supplementary Fig. 1c). Consistent with the increase in partially differentiated colonies, ExEn markers (*Gata6*, *Foxa2*, *Sox17*, *Afp*, *Ihh*, *LamininB1* and *Dab2*) were significantly upregulated in Dax1 KD cells as shown by quantitative reverse transcriptase–PCR (qRT–PCR) analysis (Fig. 1f). Also, there was a weak induction of mesoderm (*Flk1*) and TE markers (*Hand1*, *Cdx2* and *Eomes*), but not of the neuroectoderm (*Sox1* and *Nestin*) and other mesodermal markers (*Brachyury* and *Goosecoid*). Nonetheless, expression of pluripotency-associated transcripts were maintained (Fig. 1g). Immunostaining revealed that the number of Gata6-positive cells increased, but Oct4 expression appeared to be comparable in Dax1 KD and Luc KD cells (Fig. 1h).

To rule out shRNA off-target effects, we transfected an expression vector containing Dax1 cDNA (Dax1 OE) into Dax1 KD-5 ESCs. Because cDNA does not contain the 3′ UTR of Dax1, the Dax1 KD-5 construct does not affect this exogenous transcript (Supplementary Fig. 1a,d). Re-expression of Dax1 could completely rescue the upregulation of ExEn markers in Dax1 KD cells (Supplementary Fig. 1e), indicating that the observed phenotypes are specifically due to loss of Dax1 function.

Furthermore, stable Dax1 KD were also generated in an Oct4:GFP reporter iPSC line. We found that Oct4:GFP was expressed in Dax1 KD iPSCs (Supplementary Fig. 1f), whereas Gata6-positive cells increased and were mainly Nanog^low^ cells (Supplementary Fig. 1g). A colony-forming assay and qRT–PCR analysis for pluripotency and differentiation markers also produced consistent results with that of Dax1 KD ESCs (data not shown). Collectively, these results suggest that a large number of, if not all, Dax1 KD ESCs/iPSCs can maintain undifferentiated states, despite an increased differentiation propensity and reduced self-renewal efficiency.

### Dax1 KD ESCs retain multilineage differentiation potential

**Figure 2:**
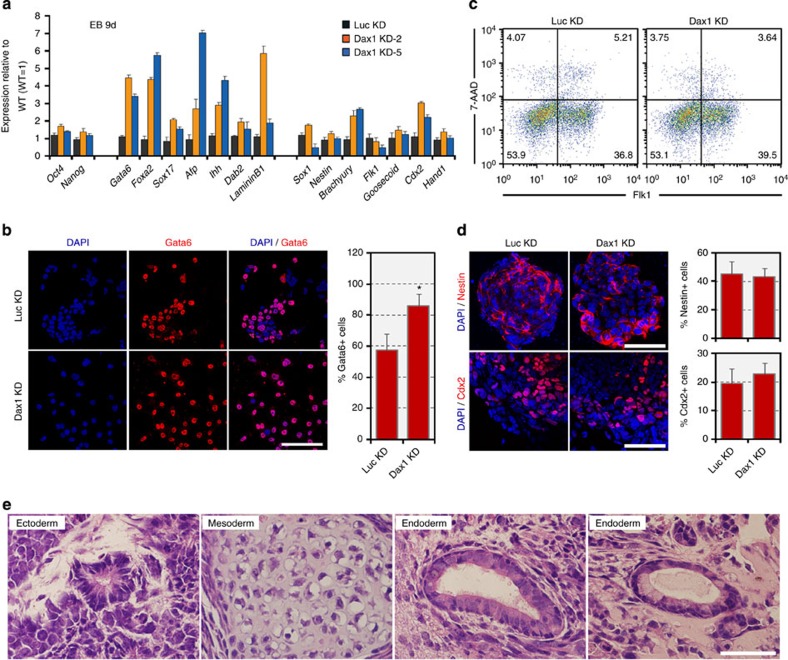
Dax1-knockdown ESCs retain multilineage differentiation potential. (**a**) qRT–PCR analysis of gene expression in the indicated lines after 9 days of EB differentiation. All data are normalized to *Gapdh* and shown relative to WT ESCs (set at 1.0). Data are represented as mean±s.d.; *n*=3. (**b**) Enhanced ExEn differentiation is observed in Dax1 KD ESCs. Monolayer cultures were treated with RA (0.1 μM) for 4 days and costained with Gata6 and DAPI. Scale bar, 100 μm. Proportions of Gata6+ cells are shown in the bar graph next to the images. Data are represented as mean±s.e.m. (*n*=3). *P<0.05. *P* values were calculated using Student’s *t*-test. (**c**) Flow cytometric analysis of mesoderm induction, as measured by the Flk1+ population, from Luc KD (control) and Dax1 KD ESCs. Numbers in quadrants indicate the percentage of each population. (**d**) Immunofluorescent analyses of neuroectoderm (Nestin, red) and TE (Cdx2, red) induction from Luc KD (control) and Dax1 KD ESCs. Scale bar, 100 μm. Proportions of Nestin+ cells and Cdx2+ cells are shown in the bar graph next to the images. Data are represented as mean±s.e.m. (*n*=3). (**e**) Neuroectoderm, mesoderm and ExEn-derived structures are present in teratomas from Dax1 KD ESCs. Hematoxylin and eosin-stained tissue sections are shown. Scale bar, 50 μm.

To assess whether Dax1 KD affects the multilineage differentiation potential of ESCs, we measured marker gene expression in day 9 embryoid bodies (EB) derived from Dax1 KD ESCs. As shown in Fig. 2a, pluripotency genes were downregulated in Dax1 KD cells on EB differentiation similar to Luc KD cells. No consistent significant variation was detected for the transcripts of neuroectoderm, mesoderm and TE, but ExEn markers were upregulated more when compared with control cells (Fig. 2a). Next, we used lineage-specific differentiation models to clarify the effect of Dax1 KD. In the presence of low-dose retinoic acid (RA), which promotes ExEn differentiation^22^, ~85% of Dax1 KD cells were Gata6 positive, compared with ~60% of Luc KD cells (Fig. 2b). Under conditions for neuroectoderm^23^, TE^24^ or mesoderm^25^ differentiation, however, expression of lineage-specific markers did not significantly differ from those in control cells (Fig. 2c,d). Notably, Dax1 KD ESCs also had the capacity to generate teratomas consisting of all three germ-derived tissues, suggesting that these cells still maintain pluripotency *in vivo* (Fig. 2e). These data indicate that Dax1 KD ESCs retain a multilineage differentiation potential, but have an enhanced propensity for differentiating into ExEn lineages.

### Dax1 OE confers LIF-independent self-renewal on ESCs

**Figure 3:**
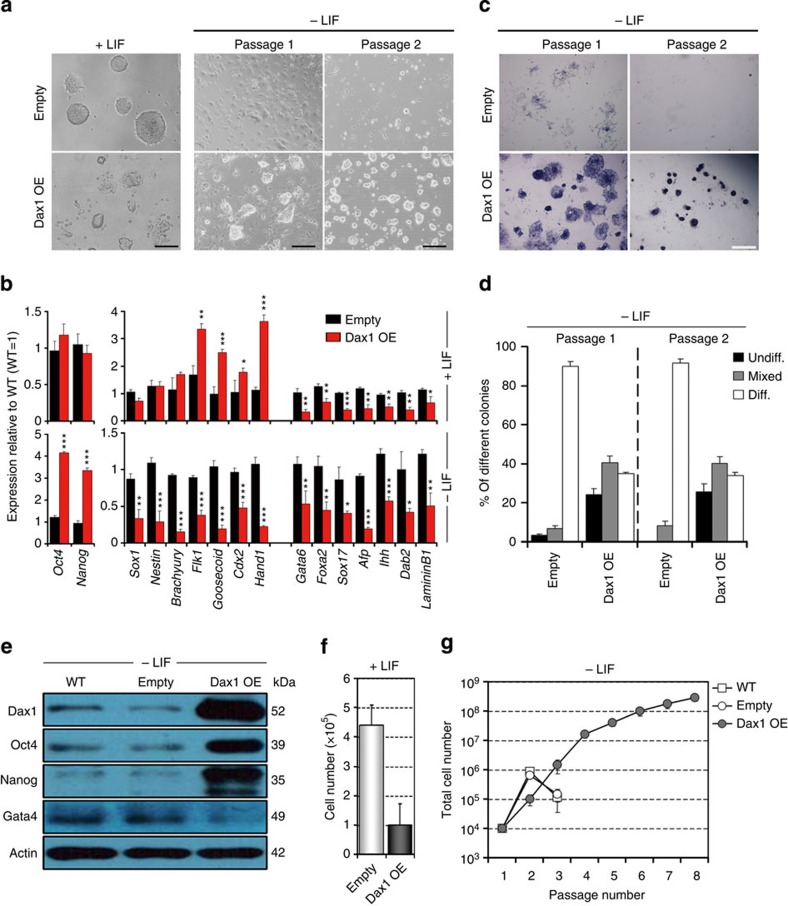
Dax1 overexpression confers LIF-independent self-renewal on ESCs. (**a**) Morphology of empty vector and Dax1-overexpressing ESCs. Cells were grown for 5 days with LIF (left), or induced to form EBs for 5 days followed by culture without LIF for two passages (right). Scale bar, 100 μm. (**b**) qRT–PCR analysis of gene expression in control (Empty) and Dax1 OE ESCs. Cells were cultured with (upper) or without LIF (lower) for 5 days. All data are normalized to *Gapdh* and shown relative to WT ESCs (set at 1.0). (**c**) Control (Empty) or Dax1 OE ESCs were passaged every 4 days without LIF and stained for AP. Scale bar, 100 μm. (**d**) Quantitation of colony types formed by cell lines shown in **c**. (**e**) Immunoblot analysis of Dax1, Oct4, Nanog and Gata4 protein in the indicated lines after 5 days of culture without LIF. β-Actin was used as an internal control. (**f**) Control (Empty) or Dax1 OE ESCs (200 cells per cm^2^ in 12-well plates) were cultured for 5 days with LIF and cells were counted. (**g**) Total cell number of the indicated lines cultured for multiple passages without LIF. Data in **b**,**d**,**f**,**g** are represented as mean±s.d.; *n*=3. *P<0.05; **P<0.01; ***P<0.001. All *P* values were calculated using Student’s *t*-test. Diff., differentiated; Undiff., undifferentiated.

To investigate the effect of Dax1 gain of function on ESC self-renewal and pluripotency, full-length cDNA for *Dax1* was cloned into the pPyCAGIP-based vector and stable Dax1 OE ESC lines were established. Under +LIF conditions, Dax1 OE cells formed more disorganized semi-differentiated-like colonies (Fig. 3a, left). qRT–PCR and immunostaining analyses showed that expression of some differentiation markers (*Flk1*, *Goosecoid*, *Cdx2* and *Hand1*) were upregulated, whereas that of the pluripotency markers were normal (Fig. 3b, upper; Supplementary Fig. 2a). After two passages without LIF, the control cells (empty vector-transfected) underwent complete differentiation. In contrast, Dax1 OE cells could form undifferentiated AP-positive colonies with a typical ESC morphology (Fig. 3a, right; Fig. 3c,d). Both mRNAs and proteins examined for lineage markers’ expression showed that, in the absence of LIF, expression of Oct4 and Nanog continued, and all differentiation markers were significantly repressed in Dax1 OE cells (Fig. 3b, lower; Fig. 3e). Cell proliferation experiments showed that despite reduced growth and increased apoptosis under +LIF conditions (Fig. 3f; Supplementary Fig. 2b), Dax1 OE ESCs could be serially passaged without LIF (Fig. 3g).

Overexpression of Dax1 at high levels creates the possibility of neomorphic effects, which may impair self-renewal of ESCs by sequestering other pluripotency factors, as reported previously^26^,^27^. We therefore used low-dose puromycin selection to generate an ESC line expressing Dax1 at nearly endogenous levels (Dax1-NE; Supplementary Fig. 2c). In contrast to the pro-differentiation phenotype induced by Dax1 OE, these cells exhibited normal ESC morphology and marker gene expression under +LIF conditions, with only a slight reduction in the AP-positive colony-forming and proliferation ability (Supplementary Fig. 2d–h). However, in the absence of LIF, Dax1-NE ESCs recapitulate the phenotype of Dax1 OE cells, including: (1) undifferentiated morphology (Supplementary Fig. 2d); (2) continuous expression of pluripotency genes; (3) decreased expression of differentiation markers (Supplementary Fig. 2e); and (4) capacity to form self-renewing colonies (Supplementary Fig. 2f,g) and to expand (Supplementary Fig. 2h). Taken together, these results indicate that Dax1 can prevent differentiation and confer LIF-independent self-renewal in ESCs, but it may not be a self-renewal-promoting factor.

### Dax1 directly inhibits *Gata6* transcription

**Figure 4:**
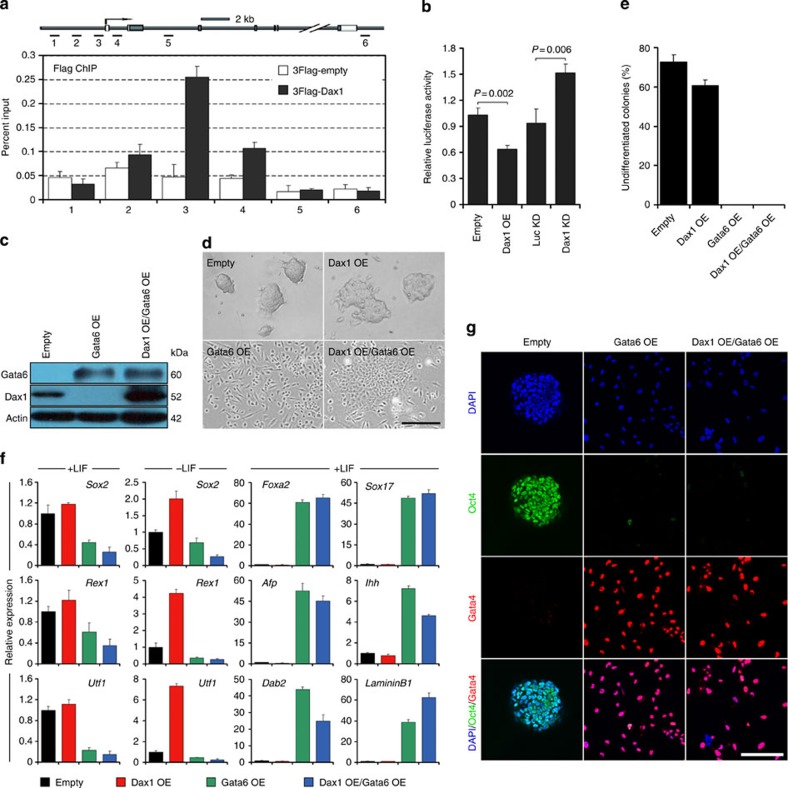
Inhibition of ExEn differentiation by Dax1 is mediated by Gata6. (**a**) ChIP-qPCR analysis of Dax1 occupancy at *Gata6* locus. Numbered grey bars indicate primer locations (upper). ESCs were transfected with 3Flag-tagged Dax1 expression vector or the 3Flag-empty vector as a control. ChIP was performed using anti-Flag antibody and qPCR analysis was performed with the primers indicated. Values are expressed as percent of input DNA (lower). (**b**) Luciferase reporter analysis shows that Dax1 OE downregulated, whereas Dax1 KD upregulated the *Gata6* promoter activity. ESCs were co-transfected with the Gata6 promoter–reporter construct and vectors as indicated. Transfected cells were cultured without LIF for 2 days and then luciferase activity was measured. Values are normalized to a *Renilla* luciferase control. The mean value of cells transfected with the Gata6 promoter–reporter construct was set at 1.0. (**c**) Immunoblot analysis of Gata6 and Dax1 proteins in control (Empty), Gata6 OE (WT ESCs transfected with Gata6 expression vector) and Dax1 OE/Gata6 OE (Dax1 OE ESCs transfected with Gata6 expression vector) cells after 5 days of culture with LIF. β-Actin was used as an internal control. (**d**) Morphology of colonies formed by control (Empty), Dax1 OE, Gata6 OE and Dax1 OE/Gata6 OE cells after 5 days of culture with LIF. Scale bar, 100 μm. (**e**) Quantitative analysis of undifferentiated colony formed by cells shown in **d**. Cells were plated at clonal density, cultured for 6 days with LIF and stained for AP. (**f**) qRT–PCR analysis of pluripotency and ExEn markers in the indicated cells cultured with or without LIF. All data are normalized to *Gapdh* and shown relative to the mean of control cells (set at 1.0). (**g**) Immunofluorescence analysis of Oct4 (green) and Gata4 (red) in the indicated lines. ESCs were transfected with each expression vector, selected for 6 days in the presence of LIF and counterstained with DAPI. Scale bar, 100 μm. Data in **a**,**b**,**e**,**f** are represented as mean±s.d.; *n*=3.

The above observations suggested that Dax1 may play a role in inhibiting ExEn differentiation, in which *Gata6* transcriptional activation is the key^11^. We thus performed chromatin immunoprecipitation (ChIP) to assess whether Dax1 binds to the *Gata6* locus *in vivo*. Significantly, an enhanced enrichment of Dax1 on *Gata6* proximal promoter was observed (Fig. 4a). To rule out other pluripotency factors that may mediate Dax1 binding to the *Gata6* promoter, we co-transfected 3Flag-Dax1 expression vector and the *Gata6* proximal promoter fragment into 293FT cells. ChIP-qPCR analysis validated that Dax1 specifically bound to this DNA segment (Supplementary Fig. 3a).

To determine whether the binding was associated with the regulation of *Gata6* transcription, we performed a luciferase assay. Data showed that *Gata6* promoter activity was repressed by Dax1 OE and increased by Dax1 KD (Fig. 4b). Likewise, Dax1 OE effectively repressed *Gata6* promoter activity in RA-treated differentiated cells (Supplementary Fig. 3b), in which Gata6 expression was activated, whereas Oct4 and Nanog were undetectable^22^. These results suggest that Dax1 can repress *Gata6* transcription in the absence of other pluripotent factors. To map the Dax1-binding region, we generated serial deletion Gata6 promoter/enhancer reporter constructs. The luciferase assay indicated that the Dax1-binding motif was located between −710 and −570 bp of the *Gata6* promoter (Supplementary Fig. 3c).

To verify whether Dax1-mediated Gata6 repression contributes to the ExEn differentiation defect of Dax1 OE ESCs, we compared phenotypes of Dax1 OE, Gata6 OE and Dax1 OE/Gata6 OE cells (Fig. 4c). Under +LIF conditions, Gata6 OE and Dax1 OE/Gata6 OE cells exhibited a completely differentiated morphology (Fig. 4d). No AP-positive colonies appeared in these cell cultures (Supplementary Figs 3d and 4e). mRNA and protein analyses showed that pluripotency markers were lost and ExEn lineage markers were strongly upregulated in both Gata6 OE and Dax1 OE/Gata6 OE cells (Fig. 4f,g; Supplementary Fig. 3e). These data indicate that as a downstream target of Dax1, Gata6 can compensate for ExEn differentiation defects caused by Dax1 OE.

### Dax1 and Nanog function in parallel to maintain pluripotency

Features of Dax1 strongly suggest a functional similarity to Nanog^8^,^9^,^15^. Analysis using published microarray data sets^28^,^29^ showed that, in contrast to significant downregulation after Oct4 or Sox2 KD, pluripotency genes only slightly changed with respect to expression after Dax1 or Nanog KD (Supplementary Fig. 4a), as validated by qRT–PCR (Supplementary Fig. 4b). After 72 h of Dax1 and Nanog depletion, 242 and 761 genes were differentially expressed, respectively, and 133 genes were common (Supplementary Fig. 4c). In contrast, 298 and 490 genes had ≥1.5-fold expression changes after induction of Dax1 or Nanog (Supplementary Fig. 4d). The statistically significant *P* values were corrected using the Benjamini and Hochberg false discovery rate test (FDR<0.05)^30^. Interestingly, only nine differentially expressed genes were found in both Dax1 OE and Nanog OE ESCs, suggesting that they may function independently.

**Figure 5:**
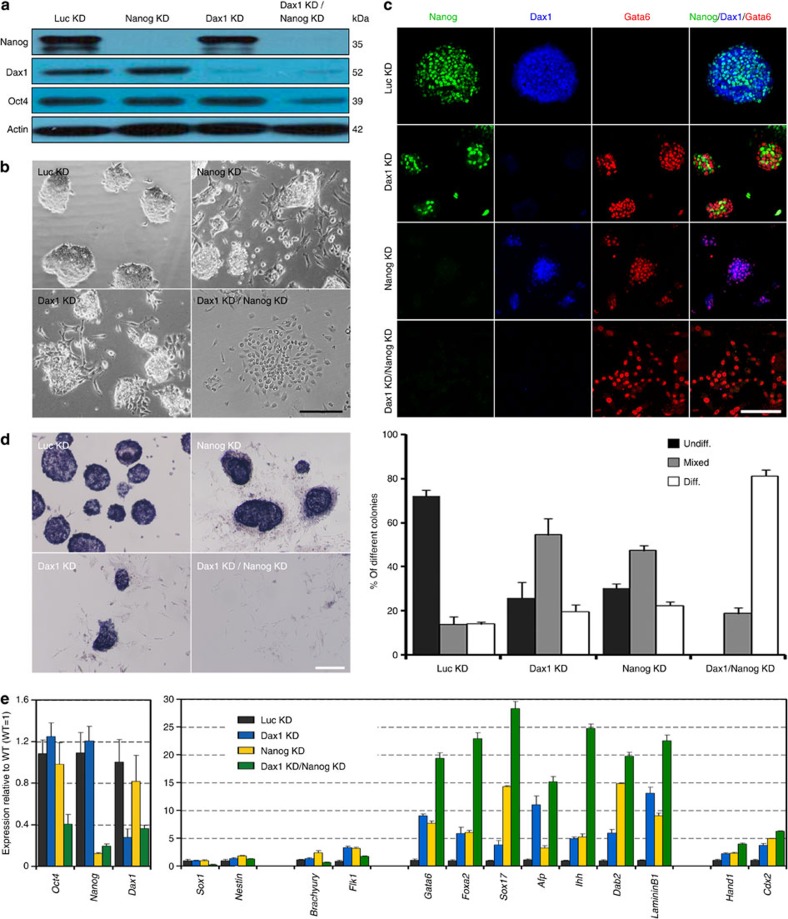
Dax1 and Nanog function in parallel and independently to regulate pluripotency of ESCs. (**a**) Immunoblot analysis of Nanog, Dax1 and Oct4 proteins in the indicated lines. ESCs were infected with luciferase shRNA (Luc KD), Nanog shRNA (Nanog KD), Dax1 shRNA (Dax1 KD) or Dax1 plus Nanog shRNA (Dax1 KD/Nanog KD) lentivirus and selected in the presence of LIF for 5 days. β-Actin was used as an internal control. (**b**) Representative morphologies of colonies formed by the indicated lines shown in **a**. Cells were cultured for 5 days with LIF. Scale bar, 100 μm. (**c**) Immunofluorescence analysis of Nanog (green), Dax1 (blue) and Gata6 (red) in the indicated lines after 5 days culture with LIF. Scale bar, 100 μm. (**d**) AP staining of colonies formed by plating the indicated shRNA-transduced cells at clonal density and culturing for 6 days in the presence of LIF (left). Scale bar, 100 μm. Percentage of colony types formed by cells is shown (right). Data are represented as mean±s.d.; *n*=3. (**e**) qRT–PCR analysis of gene expression in the indicated lines after 5 days of culture with LIF. All data are normalized to *Gapdh* and shown relative to WT ESCs (set at 1.0). Data are means±s.d.; *n*=3. Diff., differentiated; Undiff., undifferentiated.

To better understand the functional relationship between Dax1 and Nanog, we compared the phenotypic differences on Nanog KD, Dax1 KD and Dax1 plus Nanog KD (Dax1 KD/Nanog KD) in ESCs under LIF conditions (Fig. 5a). Nanog KD cells could be continuously propagated and form undifferentiated AP-positive colonies that express Oct4 and Dax1, despite an increase in differentiated-like cells and upregulation of ExEn markers (Fig. 5a–e). These phenotypes resembled those of Dax1 KD cells (Fig. 5a–e) and were consistent with the previously described phenotypes of *Nanog*^−/−^ cells^15^. In contrast to individual KD cells, Dax1 KD/Nanog KD cells were fully differentiated and could not generate AP-positive colonies. Oct4 expression was markedly downregulated, and ExEn differentiation markers were significantly further upregulated (Fig. 5a–e), indicating that a simultaneous depletion of Dax1 and Nanog induces terminal differentiation of ESCs even in the presence of LIF.

In addition to Nanog, Klf4 and Esrrb have also been shown to be dispensable for ESC self-renewal. Artificial expression of Klf4 or Esrrb is sufficient to maintain pluripotency in the absence of LIF^31^,^32^,^33^,^34^,^35^. Therefore, functional analyses of the single KD, as well as double KD of each gene with Dax1 and Nanog were performed in parallel. Two shRNA sequences, which have been previously validated^35^,^36^, were used to silence Klf4 and Esrrb, respectively (Supplementary Fig. 5a). In accordance with previous studies^35^,^37^,^38^, no obvious phenotypic changes were observed in Klf4 KD cells, whereas Esrrb KD cells displayed an increased differentiation propensity, with the upregulation of TE and neuroectoderm markers (Supplementary Fig. 5b–d). By comparison, however, Dax1/Klf4, Dax1/Esrrb, Nanog/Klf4 and Nanog/Esrrb double KD all showed significantly weaker additive effects than Dax1/Nanog KD (Supplementary Fig. 5b–d).

**Figure 6:**
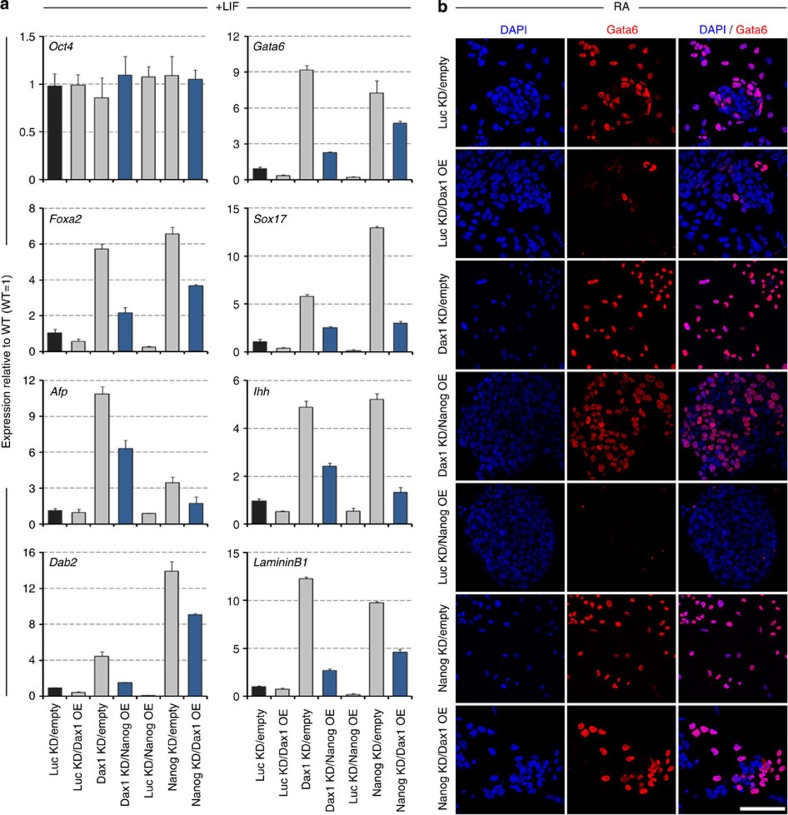
Functions of Dax1 and Nanog are partially complementary but they cannot replace each other. (**a**) qRT–PCR to measure expression of pluripotency and ExEn markers in the indicated lines after 5 days culture with LIF. All data are normalized to *Gapdh* and shown relative to WT ESCs (set at 1.0). Data are means±s.d.; *n*=3. (**b**) Immunofluorescence analysis of Gata6 in the indicated lines. Monolayer cultures were treated with RA (0.1 μM) for 4 days and counterstained with DAPI. Scale bar, 100 μm.

Next, to test whether Nanog and Dax1 are functionally redundant, we measured the expression of ExEn markers in Dax1 KD/Nanog OE and Nanog KD/Dax1 OE cells. Immunoblotting analysis showed that both Dax1 and Nanog were comparably overexpressed after transfection (Supplementary Fig. 6a,b). Under +LIF conditions, Dax1 could not completely rescue increased expression of ExEn markers induced by Nanog KD. Also, Nanog could not completely rescue the effect of Dax1 KD (Fig. 6a). Under RA-induced differentiation conditions, individual Dax1 OE or Nanog OE effectively inhibited the expression of Gata6 and formed undifferentiated AP-positive colonies. In contrast, both Dax1 KD/Nanog OE and Nanog KD/Dax1 OE cells could not completely suppress Gata6 upregulation, although they partially retained the capacity to form AP-positive colonies (Fig. 6b; Supplementary Fig. 6c). These results indicate that functions of Dax1 and Nanog are partially complementary but cannot replace each other, even though they are overexpressed.

Collectively, these data suggest that Nanog is not a downstream effector of Dax1, and vice versa. These two factors most likely function in a parallel and cooperative manner to maintain pluripotency.

### Dax1 is indispensable for self-renewal of Nanog^low^ ESCs

ESCs have been reported to fluctuate between a Nanog^high^ and a Nanog^low^ state^15^. In contrast, Dax1 was uniformly expressed in ESCs (Supplementary Fig. 7a). Nanog KD could induce Gata6 expression in Dax1^+^ cells, whereas Dax1 KD induced Gata6 derepression mainly in Nanog^−^ cells and in only a minority of Nanog^+^ cells (Supplementary Fig. 7b–d). In addition, fluorescence-activated cell sorting (FACS)-sorted Nanog^low^ ESCs had reduced expression of *Klf4* and *Esrrb* but relatively normal expression of *Oct4* and *Dax1* (Supplementary Fig. 7e). These results are consistent with the previous findings that *Klf4* and *Esrrb* are direct downstream targets of Nanog^33^,^34^, and suggest that Dax1, but not Klf4 or Esrrb, is required for preventing Nanog^low^ ESCs from differentiating. To confirm this, we established stable Dax1 KD in Nanog:GFP reporter ESCs (Supplementary Fig. 7f).

**Figure 7:**
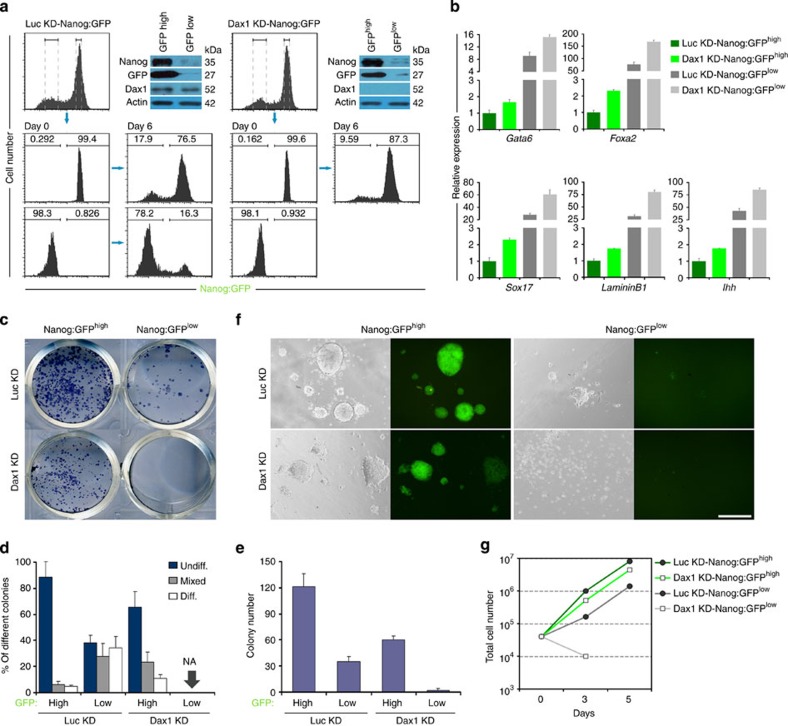
Dax1 is indispensable for self-renewal of Nanog^low^ ESC. (**a**) Luc KD- and Dax1 KD-Nanog:GFP cells were sorted into Nanog:GFP^low^ and Nanog:GFP^high^ populations (day 0). Cells were cultured in the presence of LIF for 6 days and FACS analyses were repeated. Number given is the percentage of cells in each of the indicated gates. Immunoblot analyses confirmed that GFP fluorescence reflected Nanog expression and Dax1 was efficiently silenced in both sorted Dax1 KD-Nanog:GFP populations. (**b**) qRT–PCR to measure expression of ExEn markers in the indicated FACS-purified cells. All data are normalized to *Gapdh* and shown relative to the mean of Luc KD-Nanog:GFP^high^ cells (set at 1.0). Data are means±s.d.; *n*=3. (**c**) The indicated FACS-sorted cells (1 × 10^3^ cells per cm^2^ in 12-well plates) were cultured for 6 days with LIF and stained for AP. (**d**) Percentage of colony types formed by cells shown in **c**. Data are means±s.d.; *n*=3. NA, not available. (**e**) Total colony number counts after plating the indicated FACS-sorted cells at a density of 1 × 10^3^ cells per cm^2^ in 12-well plates and culturing for 6 days with LIF. Data are means±s.d.; *n*=3. (**f**) Morphology and GFP fluorescence of the indicated FACS-sorted cells cultured with LIF. Scale bar, 100 μm. (**g**) Growth curves of the indicated FACS-sorted cells cultured with LIF. Diff., differentiated; Undiff., undifferentiated.

In agreement with previous reports^15^, GFP^high^ (green fluorescent protein) and GFP^low^ populations sorted from control (Luc KD) cells were interconvertible, although GFP^low^ cells showed obviously reduced ability of self-renewal and proliferation (Fig. 7). Comparatively, Dax1 KD-Nanog:GFP^high^ populations exhibited a higher self-renewal efficiency than Luc KD-Nanog:GFP^low^ cells. However, upregulated ExEn marker expression as well as reduced colony-forming and proliferation abilities were observed in contrast to control Nanog:GFP^high^ cells (Fig. 7b–g), indicating a relatively increased differentiation propensity in Dax1 KD-Nanog^high^ cells. In contrast, Dax1 KD-Nanog:GFP^low^ populations had more dramatically upregulated ExEn transcripts and were hardly expanded (Fig. 7b–g), indicating that Dax1 KD-Nanog^low^ cells cannot survive and/or self-renew. Accordingly, Dax1 KD-GFP^high^ cells regenerated GFP^low^ cells, but GFP^low^ cells could not give rise to GFP^high^ cells (Fig. 7a,f). These data suggest that Dax1 is not only essential but also sufficient for stabilizing ESC pluripotency during the dynamic transition between Nanog^high^ and Nanog^low^ states.

### Dax1 is required for full somatic cell reprogramming

**Figure 8:**
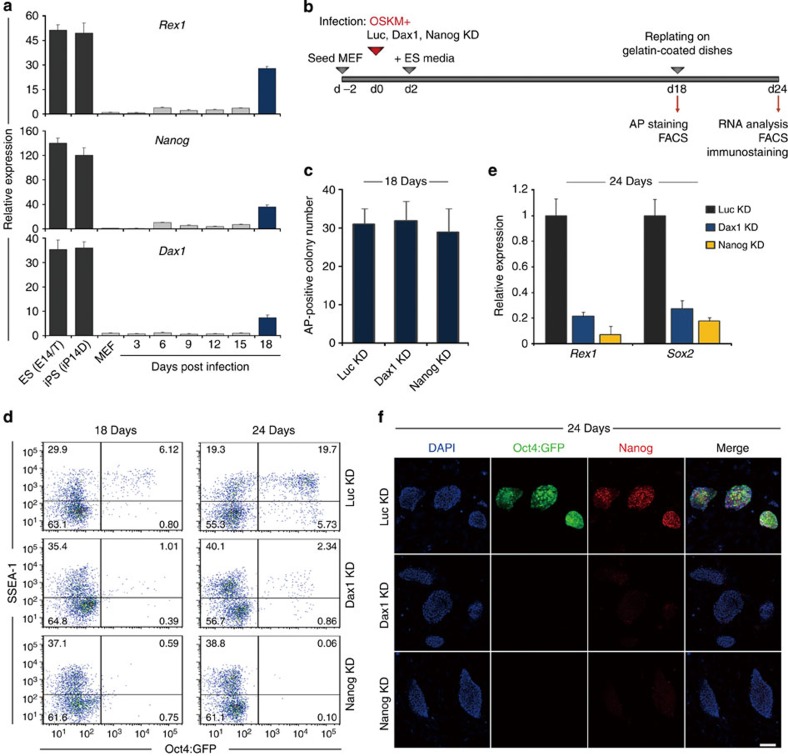
Dax1 and Nanog are both required for full reprogramming to induce pluripotency. (**a**) qRT–PCR analysis of the endogenous *Rex1*, *Nanog* and *Dax1* mRNA expressions during the course of reprogramming. All data are normalized to *Gapdh* and shown relative to the mean of MEFs (set at 1.0). Data are means±s.d.; *n*=3. (**b**) Scheme and strategy for functional studies of Dax1 and Nanog in reprogramming. (**c**) Oct4-GFP MEFs were infected with the TetO-4F2A encoding Oct4, Sox2, Klf4 and c-Myc (OSKM) and M2rtTA lentiviruses in combination with luciferase shRNA (Luc KD), Dax1 shRNA (Dax1 KD) or Nanog shRNA (Nanog KD) lentivirus. The number of AP^+^ colonies was counted 18 days after induction. Data are means±s.d.; *n*=3. (**d**) Flow cytometric analysis of Oct4-GFP reporter activity and SSEA-1 expression in the respective lentiviral-infected Oct4-GFP MEF-derived cells 18 and 24 days after induction. Numbers in quadrants indicate the percentage of each population. (**e**) qRT–PCR analysis of the endogenous *Sox2* and *Rex1* mRNA expression in the respective lentiviral-infected Oct4-GFP MEF-derived cells 24 days after induction. All data are normalized to *Gapdh* and shown relative to the mean of OSKM+Luc KD cells (set at 1.0). Data are means±s.d.; *n*=3. (**f**) Immunofluorescence analysis of Oct4-GFP (green) and Nanog (red) in the indicated lentiviral-infected Oct4-GFP MEF-derived cells 24 days after induction. Cells were counterstained with DAPI (blue). Scale bar, 50 μm. d, day.

Given the importance of Dax1 in pluripotency maintenance, we then asked whether it is necessary for efficient somatic cell reprogramming. Dax1 expression was absent in mouse embryonic fibroblasts (MEFs) but upregulated during iPSC generation alongside Nanog, Sox2 and Rex1 (Fig. 8a; Supplementary Fig. 8a). It has been reported that Dax1 is not in the original reprogramming factor cocktail Oct4, Sox2, Klf4 and c-Myc (OSKM)^3^. Moreover, addition of Dax1 to this quartet has not been shown to increase efficiencies^39^. However, whether Dax1 is, like Nanog^40^, required for attaining pluripotency at the final stage of the reprogramming process is unclear. Thus, we introduced Dax1 RNA interference lentivirus into Oct4-GFP MEFs^41^ together with OSKM reprogramming factors (Fig. 8b).

In our experiment, cell colonies started to emerge at day 9 post infection. After AP staining at day 18, no differences were found between Luc KD and Dax1 KD, as well as Nanog KD MEFs (Fig. 8c). As described previously^42^, rarer cells were GFP positive at this stage, indicating incomplete induced reprogramming (Fig. 8d). After replating onto gelatin-coated dishes and inducing for an additional 6 days, an estimated 25.4% of control cells were GFP positive. But in Dax1 KD cells, this ratio was <3.2%, which was approximate to that of Nanog KD cells (Fig. 8d). A significant decrease in the number of Nanog/Oct4-GFP-positive colonies and a marked reduced endogenous *Sox2* and *Rex1* expression were observed in both Dax1 KD and Nanog KD MEF-derived cells (Fig. 8e,f). Notably, these cells were SSEA-1 positive and proliferation was not adversely affected (Fig. 8d; Supplementary Fig. 8b). A similar reprogramming block was also observed when we used a clonal line of Oct4-GFP MEF-derived pre-iPSCs. This cell line expressed SSEA-1 and was able to spontaneously give rise to fully reprogrammed cells after passaging^43^. As Supplementary Fig. 8c–e showed, SSEA-1 expression was unchanged in both Dax1 KD and Nanog KD pre-iPSCs, but the GFP-positive cells appeared at a significantly lower frequency after prolonged culture, in comparison with control pre-iPSCs. These data suggest that Dax1 is not required for the initiation phase but may play a role in acquiring the authentic pluripotency at the final stage of somatic cell reprogramming.

### Relationship of Dax1 with other ESC regulators

**Figure 9:**
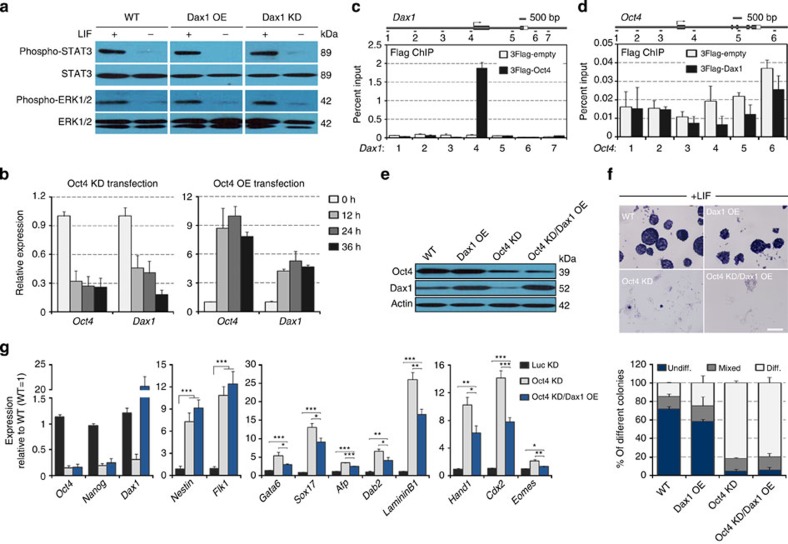
Relationship of Dax1 with other ESC regulators. (**a**) Immunoblot analysis of phospho-STAT3, total STAT3, phospho-ERK and total ERK in the indicated cells after treatment with or without LIF for 12 h. (**b**) qRT–PCR analysis of the *Dax1* mRNA expression in the Oct4 KD (left) or Oct4 OE (right) vector-transfected ESCs at 12, 24 and 36 h after transfection. All data are normalized to *Gapdh* and shown relative to the mean of untreated cells (0 h; set at 1.0). Data are means±s.d.; *n*=3. (**c**) ChIP-qPCR analysis of Oct4 occupancy at *Dax1* locus. Numbered grey bars indicate primer locations (upper). ESCs were transfected with 3Flag-tagged Oct4 expression vector or the 3Flag-empty vector as a control. ChIP was performed using anti-Flag antibody and qPCR analysis was performed with the primers indicated. Values are expressed as percent of input DNA (lower). Data are means±s.d.; *n*=3. (**d**) ChIP-qPCR analysis of Dax1 occupancy at *Oct4* locus. Data are means±s.d.; *n*=3. (**e**) Immunoblot analysis of Oct4 and Dax1 protein levels in WT, Dax1 OE, Oct4 KD and Oct4 KD/Dax1 OE (Dax1 OE ESCs transduced with Oct4 shRNA lentivirus) cells after 5 days of culture with LIF. β-Actin was used as an internal control. (**f**) AP staining of colonies formed by plating the indicated cells at clonal density and culturing for 6 days with LIF (upper). Scale bar, 100 μm. Percentage of colony types formed by cells is shown (lower). Data are means±s.d.; *n*=3. (**g**) qRT–PCR analysis of gene expression in Luc KD, Oct4 KD and Oct4 KD/Dax1 OE cells after 5 days of culture with LIF. All data are normalized to *Gapdh* and shown relative to WT ESCs (set at 1.0). Data are means±s.d.; *n*=3. *P<0.05; **P<0.01; ***P<0.001. All *P* values were calculated using Student’s *t*-test. Diff., differentiated; Undiff., undifferentiated.

Activation of STAT3, a key downstream transcription factor of the LIF/gp130 pathway, is sufficient and necessary for self-renewal in ESCs^44^. Dax1 OE can maintain ESC self-renewal without LIF, but STAT3 activation was affected by neither Dax1 OE nor Dax1 KD in the presence or absence of LIF (Fig. 9a). Suppression of the extracellular signal-regulated kinase (ERK) pathway, which is also activated by LIF signalling, promotes self-renewal of ESCs^45^. Total ERK and ERK phosphorylation were shown to be indistinguishable in WT, Dax1 OE or Dax1 KD cells (Fig. 9a). Therefore, Dax1 OE ESC self-renewal in LIF-independent cultures is an unlikely consequence of directly activating STAT3, or suppression of the ERK pathway.

Oct4 is a master regulator for ESC pluripotency. Dax1 KD or OE had no significant effect on Oct4 expression, whereas Oct4 KD reduced Dax1 and Oct4 OE increased Dax1 mRNA levels as early as 12 h post transfection (Fig. 9b). ChIP-qPCR analysis showed that Oct4 bound to the promoter of Dax1, whereas the enrichment of Dax1 on the *Oct4* gene locus (spanning from ~5 kb upstream to 2 kb downstream) was not observed (Fig. 9c,d). These results and those of previous reports^46^ support that Dax1 is a direct downstream target of Oct4.

We then examined the functional relationship between Dax1 and Oct4. Although forced expression of Dax1 conferred LIF-independent self-renewal of ESCs, Dax1 OE could not prevent differentiation induced by Oct4 KD, as shown by colony-forming assays (Fig. 9e,f). These data indicate that Oct4 persistence is required for Dax1-mediated self-renewal. Nevertheless, Dax1 OE could partially rescue Oct4 KD-induced upregulation of ExEn and TE markers’ expression (Fig. 9g), suggesting that the function of Oct4 in the maintenance of pluripotency is at least partly mediated by Dax1.

It has been reported that Dax1 binds to Oct4 and inhibits its transcriptional activity in ESCs, which in turn induces TE differentiation^26^. Consistently, our earlier findings showed that Dax1 OE led to an increased expression of TE markers (Fig. 3b). However, we also found that Dax1 KD induced the derepression of *Cdx2* and *Eomes* without altering Oct4 expression (Fig. 1f,g) and, moreover, Dax1 OE rescued Oct4 KD-induced upregulation of these transcription factors to a large extent (Fig. 9g). These data therefore suggest that Dax1 can inhibit TE differentiation independently or as a downstream effector of Oct4.

## Discussion

Dax1, a member of the orphan nuclear receptor superfamily, has been suggested to play important roles in reproductive development, sex determination, steroidogenesis and tumorigenesis^47^,^48^,^49^. Attempts to generate Dax1-knockout mice have failed^50^, suggesting an early role in embryogenesis. Loss of function of Dax1 has been reported to induce ESC differentiation, but the phenotypes arising from stable suppression of Dax1 have not been observed in these studies (Supplementary Table 1). Specifically, there was no evidence to show that Dax1 KD induced a significant downregulation of pluripotent markers. In the present study, we observed that stable Dax1 KD ESC lines can be established and maintained in culture by serial passages. Dax1 KD ESCs are prone to differentiation, but retain pluripotency despite reduced self-renewal efficiency. That Dax1 KD decreases the proliferation of ESCs suggests that Dax1-null ESCs cannot survive, which may be why several laboratories have tried but failed to create Dax1-knockout ESC lines or mice.

Dax1 OE also inhibits the proliferation of ESCs and upregulates some differentiation markers. Nevertheless, Dax1 OE ESCs can be continuously propagated without LIF and maintain self-renewal. These seemingly contradictory phenotypes suggest that Dax1 may not be a self-renewal-promoting factor. In earlier studies, Dax1 OE was reported to induce ESC differentiation because Dax1 binds Oct4 and inhibits its transcriptional activity^26^. However, we report that Dax1 OE ESCs can be established and retain normal expression of pluripotent markers. This discrepancy may be due to different expression levels of extrinsic Dax1 in ESCs. Sun *et al*.^26^ observed the phenotypes of primary transfectants after supertransfection of an episomal expression vector, which directs strong expression of extrinsic Dax1 in ESCs. In this study, we also use the episomal vector system for forced expression of Dax1. However, we obtained stable transfectants by conventional transfection and drug selection. Differentiated cells in response to Dax1 OE were gradually eliminated during the culture.

Cellular phenotypic analysis indicates that Dax1 functions similarly to Nanog in regulating pluripotency. It has been reported that Nanog and its downstream target Esrrb regulate *Dax1* transcription^27^,^51^. Our work and that of others^15^ suggest that Nanog deficiency in ESCs does not affect expression of Dax1. In contrast, Nanog expression is also persistent in Dax1 KD ESCs. The Dax1 KD phenotype can be completely rescued by Dax1, but only partially rescued by Nanog OE, and vice versa. Moreover, co-knockdown of Dax1 and Nanog shows an additive effect. If Dax1 is simply a downstream effector of Nanog (or the contrary), these results cannot be explained. In fact, high-throughput experiments have also revealed that Dax1 and Nanog are two independent target ‘hubs’ within the ESC pluripotency network^19^. Thus, we suggested that Dax1 and Nanog are functionally independent, partially complementary, but ultimately irreplaceable.

**Figure 10:**
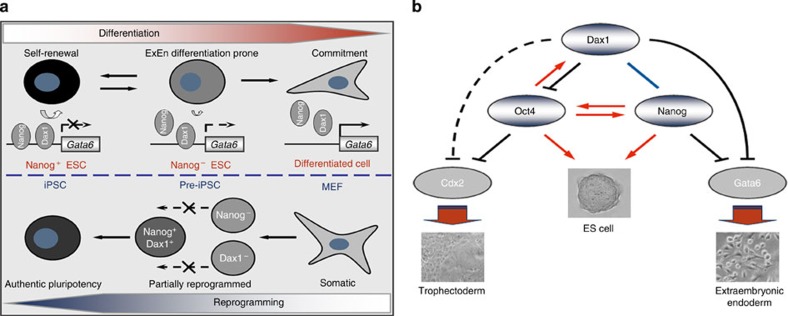
Dax1 and Nanog offer double insurance for stabilizing ESC and induced pluripotency. (**a**) Schematic representation of the functional roles of Dax1 and Nanog in stabilization of ESC and induced pluripotency. See the text for details. (**b**) Proposed model of the interplay of Dax1, Oct4 and Nanog in regulating ESC self-renewal and differentiation into either the TE or ExEn. Red indicates positive regulation; black indicates repression; and blue indicates parallel function. It is unclear whether Dax1 regulates Cdx2 directly (dashed black line). See the text for details.

Although it is not among the collection of genes for somatic cell reprogramming, Nanog has been proven to be key to the acquisition of both embryonic and induced pluripotency^40^. However, Nanog expression is heterogeneous and dynamic in ESCs/iPSCs. Fluctuating Nanog expression will inevitably lead the gene regulatory network centred around Nanog to be broken and reconstructed repeatedly, and thereby cause fluctuations in cellular function and potential. Thus, maintenance of pluripotency and self-renewal in the absence of Nanog is worthy of understanding. A logical corollary is that different populations of stem cells have networks comprised of different elements but with similar functions to Nanog^52^. Our findings provide experimental evidence not only to support this theoretical model, but also to extend recent reports establishing critical roles for Nanog in true iPSC generation. We therefore proposed a ‘double-insurance’ mechanism that Dax1 and Nanog cooperate to stabilize the ESC and induced pluripotency (Fig. 10a). In ESCs, each factor is necessary but neither Dax1 nor Nanog alone is sufficient to maintain an optimal pluripotent state. Nanog^+^/Dax1^+^ cells possess the most prominent self-renewal efficiency; Nanog^−^/Dax1^+^ ESCs have reduced self-renewal efficiency and an increased ExEn differentiation propensity, whereas Nanog^−^/Dax1^−^ ESCs irreversibly lose pluripotency. As a result, Dax1 is indispensable for keeping Nanog^−^ populations from differentiating. In producing iPSCs, Dax1 and Nanog are both needed to obtain authentic pluripotency at the final stage. Lack of each blocks the transition of reprogramming cells from the intermediate to the maturation phase.

Both Dax1- and Nanog-deficient ESCs have an increased differentiation propensity for ExEn cells, in which Gata6 is the critical regulator^11^,^53^. Co-knockdown of Dax1 and Nanog induces ExEn commitment, which might be due to full activation of Gata6. The combined effect of Dax1 and Nanog suggests that these two factors may cooperate to control expression of Gata6, and thereby determine the degree of pluripotency in ESCs (Fig. 10a). At this time, no direct evidence for the repression of *Gata6* by pluripotent factors has been reported^54^. Our observations show that Dax1 blocks ExEn differentiation by transcriptional repression of *Gata6*. Early studies suggest that Dax1 may repress gene expression by recruiting co-repressors or by interacting with stem-loop structures^55^,^56^. We identified a putative Dax1-binding site that is located −710 to −570 bp upstream from the transcription start site of the *Gata6* gene. Sequence analysis indicates that there is no proper stem-loop structure that can be formed by the DNA. Thus, other mechanisms may contribute to Dax1-mediated transcriptional regulation of *Gata6*. Nanog has also been reported to bind to the proximal region of the *Gata6* promoter^14^,^57^, indicating the tight control of this crucial target gene by Dax1 and Nanog in ESCs.

Oct4 is the core regulator of ESC pluripotency. Oct4 OE can also upregulate the expression of Gata6^12^. However, because Oct4 expression is not affected by Dax1 KD and Gata6 is only weakly induced by Oct4 OE^11^, we suggest that the predisposition of Dax1 KD ESCs to differentiate into ExEn is Oct4 independent. Both Dax1 and Nanog are regulated by Oct4 at the transcriptional level^46^,^58^, and they also form negative- and positive-feedback loops with Oct4, respectively^27^,^59^. This model would balance expression and functional activity of Oct4 (Fig. 10b), and thereby ensure the ability of ESCs to self-renew and appropriately respond to differentiation signals.

The interaction between Oct4 and Cdx2, which form a complex for the reciprocal repression of their target genes in ESCs, has been shown to regulate TE lineage determination^13^. It was reported that a 50% decrease in Oct4 induces TE differentiation^12^. However, recent studies indicate that reduced Oct4 expression (up to sevenfold) directs a more robust pluripotent state^60^,^61^. This discrepancy may suggest that Oct4 is not an exclusive factor responsible for inhibiting TE differentiation. Here we observed that both KD and OE of Dax1 lead to derepression of Cdx2 expression, but the mechanisms involved may be different. Given that Dax1 OE induced Cdx2 upregulation is due to Oct4 activity inhibition^26^, Dax1 KD-induced derepression of Cdx2 and Dax1 OE rescuing the Oct4 KD-induced Cdx2 upregulation should not be caused by functional inhibition of Oct4. Thus, these data suggest that Cdx2 can be regulated in an Oct4-independent manner, and conversely, Dax1 is required, at least partially, for Oct4-mediated Cdx2 repression. We suggest that Dax1 may play an important role in TE differentiation by interacting with Cdx2 and/or Eomes (Fig. 10b). Future studies should elucidate such mechanisms.

In conclusion, our findings reveal functional relationships of Dax1 with Nanog and Oct4, and provide new insights into the molecular mechanisms underlying somatic cell reprogramming and the dynamic equilibrium of pluripotent states. Mouse and human ESCs fluctuate between the primed state, in which they are ready for lineage commitment, and the naive/ground state, in which they have enhanced self-renewal efficiency and even totipotent properties^60^,^62^,^63^,^64^. Understanding the mechanism for stabilizing the pluripotent state is required for exploiting the full potential of ESCs and creating high-quality iPSCs. Further experiments will be needed to demonstrate the exact biological role of Dax1 in stem cell state determination and elucidate precisely the interconnection between Dax1 and other pluripotency-regulating factors.

## Methods

### Plasmid construction

pLL3.7 vector (provided by Luk Van Parijs) was modified by replacing the EGFP gene with the zeocin or hygromycin-resistance gene. All shRNA-targeting sequences (Supplementary Table 5) were designed and BLASTed to ensure specificity. The oligonucleotides encoding target shRNA were cloned as described before^65^. pPyCAGIP expression vector (a gift from Ian Chambers) was modified by replacing the IRES-pac cassette with the 2A sequence hygromycin-resistance gene. The new vector was named pCAG-2AH. The full-length open reading frames of *Dax1*, *Nanog* and *Gata6* were PCR amplified from mouse ESC cDNA using KOD-Plus- (TOYOBO) and cloned into pGEM-T Easy (Promega). After DNA sequence verification, the open reading frames were subcloned into pPyCAGIP or pCAG-2AH. For construction of Flag-tagged expression vectors, the 3 × FLAG fragments were obtained from p3 × FLAG-CMV plasmid via PCR, and subcloned in-frame into the respective expression vectors. *Gata6* promoter (positions −2,097 to +13, −1,007 to +13, −863 to +13, −710 to +13, −570 to +13 and −280 to +13) and enhancer (positions +247 to +1,539) fragments were amplified by PCR from mouse genomic DNA and inserted into pGL3-basic vector (Promega). The primers for construction are listed in Supplementary Table 6. The DNA sequences of these plasmids are available on request.

### Cell culture

Mouse ESC lines and iPSCs (Supplementary Table 2), including R1 (American Type Culture Collection), CCE (Stemcell Technologies), E14/T (provided by Ian Chambers), Nanog:GFP (a gift from Shaorong Gao) and iP14D (a gift from Qi Zhou), and their derivatives were cultured on 0.2% gelatin-coated plates in ESC medium (DMEM, 5% ES cell-qualified fetal bovine serum (FBS), 15% KSR, 2 mM GlutaMAX, 1 mM sodium pyruvate, 0.1 mM non-essential amino acids, 0.1 mM β-mercaptoethanol (all from Invitrogen) and 10 ng ml^−1^ LIF (Milipore)). Cells were routinely propagated by trypsinization and replated every 2–3 days, with a split ratio of 1 in 8. 293FT cells (Invitrogen) were maintained in DMEM supplemented with 10% FBS. All cell cultures were maintained at 37 °C with 5% CO_2_.

### ESC transfection

The plasmid DNA was transfected using Lipofectamine 2000 (Invitrogen) according to the manufacturer’s instructions. Cells were cultured in the presence of 2 μg ml^−1^ puromycin (Invitrogen) or 200 μg ml^−1^ hygromycin B (Invitrogen) for the indicated number of days. For stable transfection, resistant colonies were pooled and expanded for further analysis.

### ESC differentiation

To form EBs, ESCs were trypsinized to single-cell suspensions, plated at a density of 5 × 10^4^ cells per ml in petri dishes and cultured in LIF-deficient ESC medium for the indicated number of days. The medium was changed at 2-day intervals.

For the ExEn differentiation, cells in monolayer were treated with 0.1 μM RA^22^,^66^. Four days later, cells were fixed with paraformaldehyde and stained with Gata6, Gata4 or Foxa2 antibody.

Induction of mesodermal cells was performed as described^25^ with minor modifications. Briefly, 3 × 10^4^ ESCs were seeded in each well of collagen IV-coated six-well cluster dishes (BD Biosciences) and incubated for 4 days in α-MEM with 10% FBS and 50 μM β-mercaptoethanol. Cells were collected with cell dissociation buffer (Invitrogen) and analysed for expression of Flk1 by flow cytometry.

Neuroectoderm differentiation was performed as reported^23^. Briefly, 1.5 × 10^5^ ESCs were cultured in six-well plates in ESC medium. After 24 h of culture, the ESC medium was replaced by differentiation medium (advanced DMEM/F12 and neurobasal media (1:1), 1 × N2, 1 × B27, 2 mM GlutaMAX, 1 mM sodium pyruvate, 0.1 mM non-essential amino acids, 0.1 mM β-mercaptoethanol, 25 μg ml^−1^ insulin and 50 μg ml^−1^ bovine serum albumin). Six days later, cells were fixed with paraformaldehyde and stained with anti-Nestin antibody.

Trophoblast stem (TS) cell differentiation assays were performed as previously described^24^, 2 × 10^5^ ESCs were cultured in six-well plates in ESC medium without LIF for 42 h. Wnt3a (50 ng ml^−1^) was subsequently added to the culture. After 6 h, cells were trypsinized and replated on six-well plates (1 × 10^4^ cells per well) in TS medium. The TS medium consisted of 70% MEF-conditioned medium, 30% RPMI1640 (Invitrogen) supplemented with 10% FBS, 1 mM sodium pyruvate, 0.1 mM non-essential amino acids, 0.1 mM β-mercaptoethanol, 2 μg ml^−1^ sodium heparin (Sigma) and 25 ng ml^−1^ recombinant fibroblast growth factor 4 (Sigma). Six days later, cells were fixed with paraformaldehyde and stained with the anti-Cdx2 antibody.

### Lentiviral production

Lentiviral vector, pSPAX2 and pMD2G (4:3:1) were co-transfected into 293FT cells by calcium phosphate transfection. Briefly, 293FT cells were grown to 80% confluence in DMEM/10% FBS. Then, 12.5 μg total plasmids were mixed with 50 μl CaCl_2_ (2.5 mM) and diluted with 1/10 TE buffer (1 mM Tris–HCl, 0.1 mM EDTA, pH 7.6) to a final volume of 0.5 ml. The mixture was added dropwise to 0.5 ml 2 × HeBS (HeBS contains 0.28 M NaCl, 0.05 M HEPES, 1.5 mM Na_2_HPO_4_, pH 7.0) and mixed. After 1 min, the solution was mixed again and added dropwise into the media. Then, 12 h later, the medium was changed and virus was collected after a subsequent 48–72 h cultivation. Viral supernatant was centrifuged (10 min at 300 *g*; 4 °C) to remove cell debris. Viral particles were concentrated by ultracentrifugation at 70,000 *g* for 2 h.

### Lentiviral infection of ESCs

ESCs were trypsinized and infected in suspension by the viral supernatant along with polybrene (4 μg ml^−1^; Sigma) for 30 min at 37 °C. Cells were then plated at a density of 1 × 10^4^ cells in 24-well plates. After 48 h, cells were trypsinized, replated at 1 × 10^4^ cells per gelatin-coated 10-mm dish and cultured in ESC medium supplemented with 15 μg ml^−1^ zeocin (Invitrogen) or 200 μg ml^−1^ hygromycin B (Invitrogen) for 7 days.

### Somatic cell reprogramming

iPSCs were induced as previously described^67^,^68^. Briefly, MEFs (passage 2–4) were seeded in gelatin-coated six-well plates at a density of 1 × 10^5^ cells per well (day −2). Cells were infected with TetO-4F2A and M2rtTA lentivirus together with pLL3.7-based lentivirus for shRNAs (day 0). Infections were performed in the presence of 8 μg ml^−1^ polybrene and 1 μg ml^−1^ doxycycline. Two days later, culture medium was replaced by ESC medium supplemented with doxycycline (1 μg ml^−1^) and changed daily. On day 18, cells were trypsinized and replated onto gelatin-coated plates at a density of 5 × 10^3^ cells per cm^2^. Six days later (day 24), cells were collected for analysis.

### Cell proliferation and colony formation assay

For proliferation assay, cells were plated at a density of 200 cells per cm^2^ in gelatin-coated dishes and cultured in the presence or absence of LIF. Cells were counted at the indicated time points. Viable cells were determined by Trypan blue exclusion and manually counted using a haemocytometer under light microscopy.

For colony formation assay, ESCs were plated at clonal density (50 cells per cm^2^) and cultured in the presence (1 ng ml^−1^) or absence of LIF. After 6 days, AP was measured with a BCIP/NBT alkaline phosphatase detection kit (Beyotime) according to the manufacturer’s instructions. Colonies were scored in three categories: undifferentiated, mixed (partially differentiated) and differentiated.

### Immunofluorescence

Cells were fixed in 4% paraformaldehyde for 20 min at 4 °C and permeabilized with 0.1% Triton X-100 for 15 min, followed by blocking with 10% FBS/phosphate-buffered saline (PBS) for 30 min. Cells were stained overnight with primary antibodies at 4 °C, followed by the appropriate secondary antibodies detecting mouse and rabbit IgG. A list of antibodies and dilution ratios is available in the Supplementary Table 4. Cell nuclei were visualized by staining with DAPI (4',6-diamidino-2-phenylindole). Images were captured with a confocal microscope (SP1, Leica Microsystems) or inverted biological microscope (Olympus, IX71).

### Protein extraction and immunoblotting

To obtain whole-cell extracts, cells were washed with PBS and incubated for 20 min in cold lysis buffer containing freshly added protease inhibitors (Beyotime). Protein concentrations were determined using BCA protein assay (Beyotime). Total protein (10 μg) was separated by SDS–PAGE and transferred to polyvinylidene difluoride membranes (Millipore). Membranes were probed with specific primary antibodies, antibody–protein complex detected by horseradish peroxidase-conjugated secondary antibodies and enhanced chemiluminescence (ECL) exposed (Pierce). Representative full-length images of immunoblots are shown in Supplementary Fig. 9.

### Flow cytometry analysis

Cells were collected using cell dissociation buffer (Invitrogen). To examine the expression of Flk1, cells were resuspended at ~5 × 10^5^ cells per ml in PBS. Phycoerythrin-conjugated anti-Flk1 antibody (eBioscience) was added, cells were incubated at 4 °C for 30 min and washed in PBS. For intracellular antigens (Oct4, Nanog, Dax1 and Gata6) staining, 5 × 10^5^ cells were washed with PBS and resuspended in 1 ml Foxp3 Fixation/Permeabilization working solution (eBioscience) at 4 °C for 60 min in dark. After washing two times with Permeabilization Buffer (eBioscience), the pellet was resuspended in 100 μl 1 × Permeabilization Buffer, blocked with 2% bovine serum albumin/PBS at room temperature for 15 min followed by incubation with the recommended amount of antibody (Supplementary Table 4) at 4 °C for 60 min. Fluorochrome-conjugated secondary antibodies (Supplementary Table 4) were added for 20 min at room temperature. After washing twice with Permeabilization Buffer, cells were analysed using a FACS Calibur or FACS Aria (Becton Dickinson).

### Teratoma formation

Six-week-old male nude mice with BALB/c genetic background were used in the experiments. Mice were housed in specific pathogen-free conditions. All of the animal experiments were approved by the Animal Ethical and Experimental Committee of Third Military Medical University. ESCs were collected, washed twice with PBS and injected subcutaneously into the posterior flanks of nude mice (2 × 10^6^ cells per injection). Three mice were used for each cell line. After 4 weeks, mice were killed. Tumours were fixed with 4% formaldehyde, sectioned and stained with hematoxylin and eosin.

### Luciferase assay

Briefly, 0.5 μg of luciferase reporter was co-transfected into ESCs or 293FT cells using Lipofectamine 2000 with 0.01 μg of pRL-SV40 (Promega) as an internal control. Transfectants were lysed at 48 h after transfection and luciferase activities were measured using Dual-luciferase Reporter Assay System (Promega).

### Real-time PCR analysis

Total RNA was isolated with Trizol reagent (Invitrogen) according to the manufacturer’s recommendation. First-strand cDNA was synthesized from 1 μg of total RNA in a 10 μl reaction with oligo dT primer using the Transcriptor High Fidelity cDNA Synthesis Kit (Roche). Real-time PCR reaction was performed with the ExTaq SYBR Green Supermix (Takara) using the Eco real-time PCR System (illumina). Each PCR reaction generated a specific amplicon, as demonstrated by melting-temperature profiles of final products (dissociation curve analysis). No PCR products were observed in the absence of template. In gene expression analysis, all data were normalized to *Gapdh* and shown relative to a control sample (set at 1.0). The qPCR primers are listed in Supplementary Table 3.

### Chromatin immunoprecipitation

ChIP was performed using the ChIP Assay Kit (Upstate Biotechnology) according to the manufacturer’s instructions. Briefly, cells were crosslinked with 1% formaldehyde for 10 min at room temperature, and quenched by the 125 mM glycine. Chromatin extracts containing DNA fragments with an average size of 500 bp were immunoprecipitated using Flag antibody (Beyotime). For all ChIP experiments, qPCR analyses were performed using the Eco real-time PCR System (Illumina) and SYBR green master mix, as described above. Enrichment was calculated relative to input. The ChIP-qPCR primers are listed in Supplementary Table 7.

### Microarray analysis

Microarray data used in this study were downloaded from GEO database at NCBI ( http://www.ncbi.nlm.nih.gov/geo/query/acc.cgi?acc=GSE26520). Heatmaps were generated by Hierarchical Clustering in Cluster 3.0 and visualized using Java Treeview. Differentially expressed genes were identified based on the systematic analysis by global gene expression profiling of ESCs after KD or induction of series transcription factors^28^,^29^. The analysis was performed at NIA Array Analysis database ( http://lgsun.grc.nia.nih.gov/ANOVA/index.html), via pair-wise statistical comparison between microarray data by analysis of variance and FDR carried out using the Benjamini–Hochberg method^30^. Differentially expressed genes were identified according to the statistical criteria (FDR <0.05 and expression fold change ≥1.5). Overlap of differentially expressed genes was analysed using venn diagram analysis tool developed for ArrayTrack ( http://www.fda.gov/ScienceResearch/BioinformaticsTools/Arraytrack/).

### Statistical analysis

Statistical analysis was performed using the Statistical Package for Social Science (SPSS for Windows package release 13.0; SPSS, Chicago, IL). Student’s *t*-test was used to analyse statistical differences. Data in the figures were expressed as mean±s.d. or mean±s.e.m., and *P*<0.05 was considered significant. Each experiment was performed at least three times.

## Author contributions

All authors contributed to the design of the experiments. J.Z., G.L. and Y.R. performed experiments. All authors contributed to the analysis and interpretation of data. J.W. and R.J. wrote the manuscript.

## Additional information

**How to cite this article:** Zhang, J. *et al*. Dax1 and Nanog act in parallel to stabilize mouse embryonic stem cells and induced pluripotency. *Nat. Commun.* 5:5042 doi: 10.1038/ncomms6042 (2014).

## Supplementary Material

Supplementary Figures, Tables and References Supplementary Figures 1-9, Supplementary Tables 1-7 and Supplementary References
