# Supplementary material for: Dax1 and Nanog act in parallel to stabilize mouse embryonic stem cells and induced pluripotency
Source: Nat Commun. 2014 Oct 6;5:5042. doi: 10.1038/ncomms6042 (PMC4205889; doi:10.1038/ncomms6042)
Supplement: Supplementary Figures, Tables and References — Supplementary Figures 1-9, Supplementary Tables 1-7 and Supplementary References [file ncomms6042-s1.pdf]

Supplementary figures and legends

Supplementary Figure 1

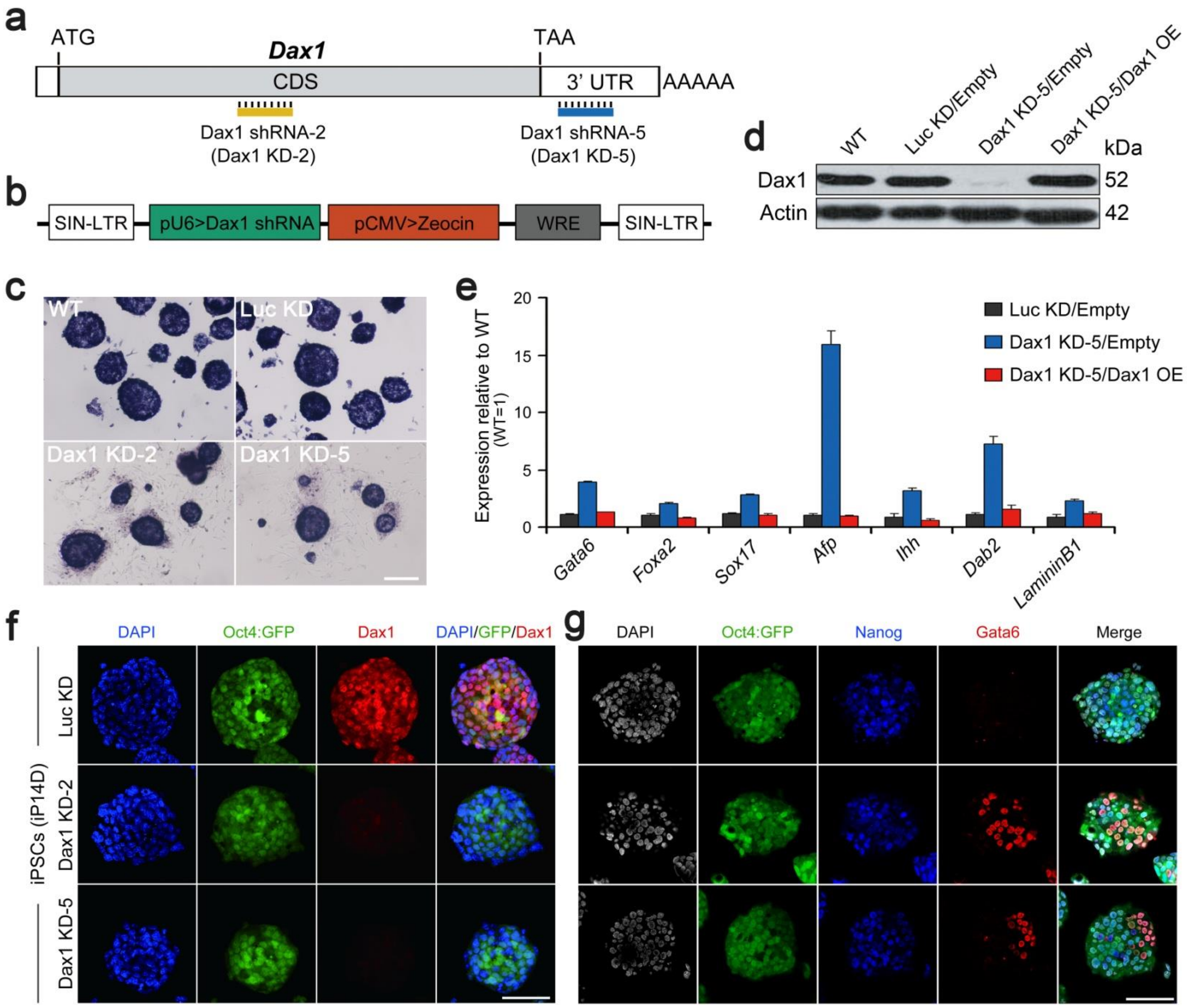

Supplementary Figure 1. Phenotypes of Dax1 knockdown ESCs/iPSCs, which can be rescued by re-expression of Dax1 cDNA

(**a**) Two shRNA constructs (Dax1 KD-2 and Dax1 KD-5) targeting the coding sequence (CDS) and the 3'-untranslated region (3'UTR) of *Dax1*, respectively.

(**b**) Schematic diagram of lentivirus-based Dax1 shRNA construct with the cassette of CMV-Zeocin.

(**c**) Alkaline phosphatase (AP) staining of colonies formed by plating the indicated lines at clonal density and culturing for 6 days in the presence of LIF. Scale bar, 100 μm.

(**d**) Immunoblot analysis of Dax1 protein in the indicated lines cultured with LIF. β-Actin was used as an internal control. Notice that the expression of exogenous Dax1 in Dax1 KD-5/Dax1 OE (Dax1 rescue) ESCs is comparable to the expression of endogenous Dax1 in WT and Luc KD/Empty (control) ESCs.

(**e**) qRT-PCR analysis of ExEn markers in the indicated lines. Gene expression was restored to control (Luc KD) levels in Dax1-rescued ESCs (Dax1 KD-5/Dax1 OE). All data are normalized to *Gapdh* and shown relative to WT ESCs (set at 1.0). Data are represented as mean ± SD; n=3.

(**f**) Immunofluorescence analysis of Dax1 KD in Oct4-GFP reporter iPSCs (iP14D, which carries an Oct4 promoter-driven GFP cassette as an additive transgene). Cells were cultured for 5 days with LIF, stained for Dax1 (red) and counterstained with DAPI. Scale bar, 100 μm.

(**g**). Immunofluorescence analysis of Nanog (blue) and Gata6 (red) in the indicated lines. Cells were cultured for 5 days with LIF and counterstained with DAPI. Scale bar, 100 μm.

Supplementary Figure 2

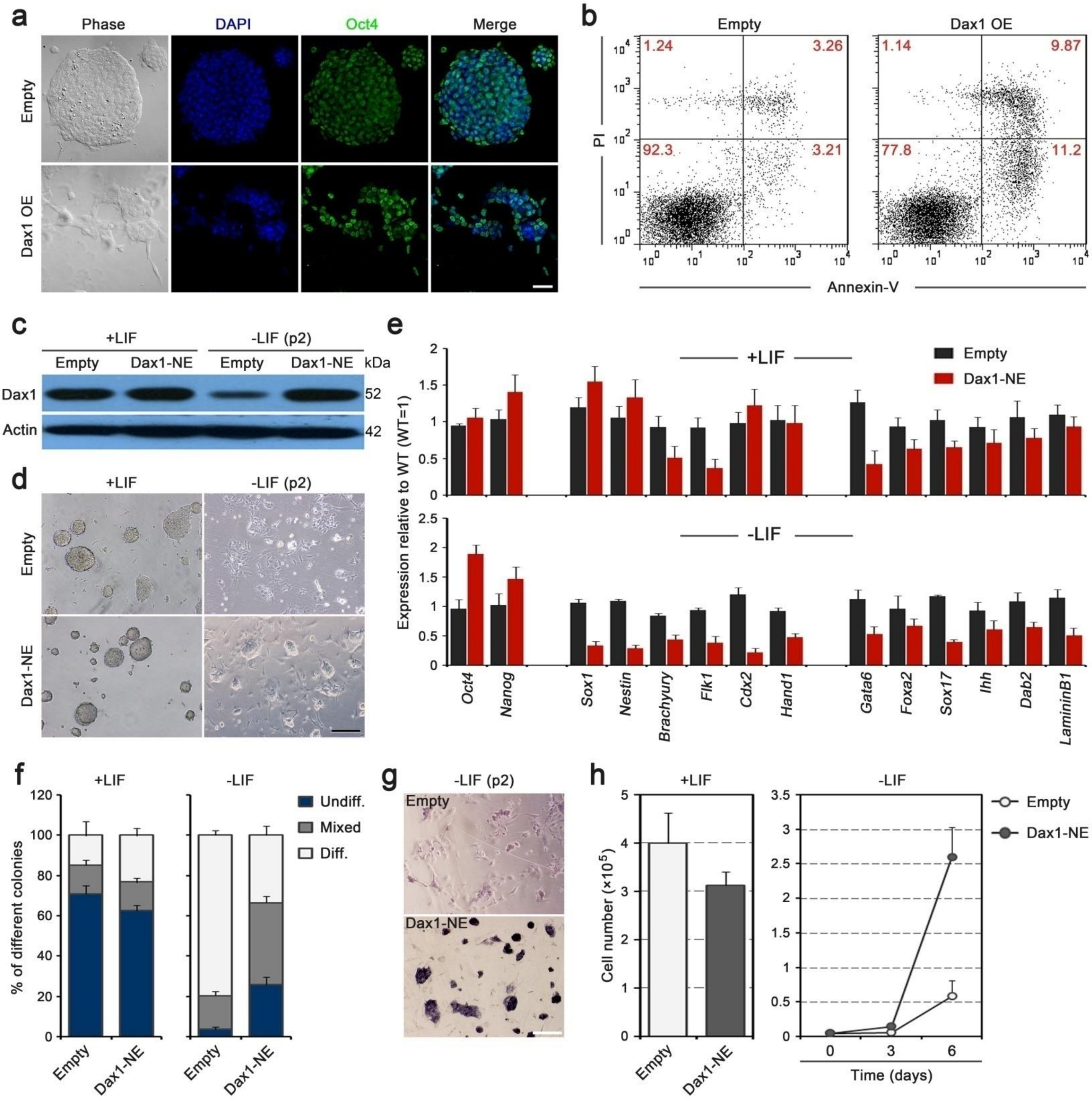

Supplementary Figure 2. Forced expression of Dax1 maintains pluripotency

(**a**) Immunofluorescent analysis of Oct4 (green) in the indicated lines. Cells were cultured with LIF for 5 days and counterstained with DAPI (blue). Scale bar, 50 μm.

(**b**) Exponentially growing ESCs were analyzed for apoptosis using Annexin-V and propidium iodide

(PI) staining and flow cytometry. Numbers in quadrants indicate the percentage of each population.

(**c**) Immunoblot analysis of Dax1 protein in control (Empty) and Dax1-NE (ESCs expressing Dax1 at nearly endogenous levels) ESC lines. Cells were cultured with LIF for 5 days, or without LIF for 2 passages. β-Actin was used as an internal control.

(**d**) Morphology of control (Empty) and Dax1-NE ESCs. Cells were cultured with LIF for 5 days, or without LIF for 2 passages. Scale bar, 100 μm.

(**e**) qRT-PCR analysis of gene expression in control (Empty) and Dax1-NE ESCs. Cells were cultured with (upper) or without LIF (lower) for 5 days. All data are normalized to *Gapdh* and shown relative to WT ESCs (set at 1.0).

(**f**) Quantitative analysis of colony formation assay in the indicated lines. Cells were plated at clonal density and cultured with or without LIF for 6 days. Colonies were fixed and stained for AP and scored as undifferentiated, mixed, or differentiated.

(**g**) Control (Empty) or Dax1-NE ESCs were cultured without LIF for 2 passages and stained for AP. Scale bar, 100 μm.

(**h**) Left: control (Empty) or Dax1-NE ESCs (1×10^3 cells per well of 12-well plates) were cultured for 5 days with LIF and cells were counted. Right: the indicated lines were plated at 5×10^3 cells per well of 12-well plates. Cell numbers were counted after 3 and 6 days.

Data in (**e**), (**f**) and (**h**) are represented as mean ± SD; n = 3.

Supplementary Figure 3

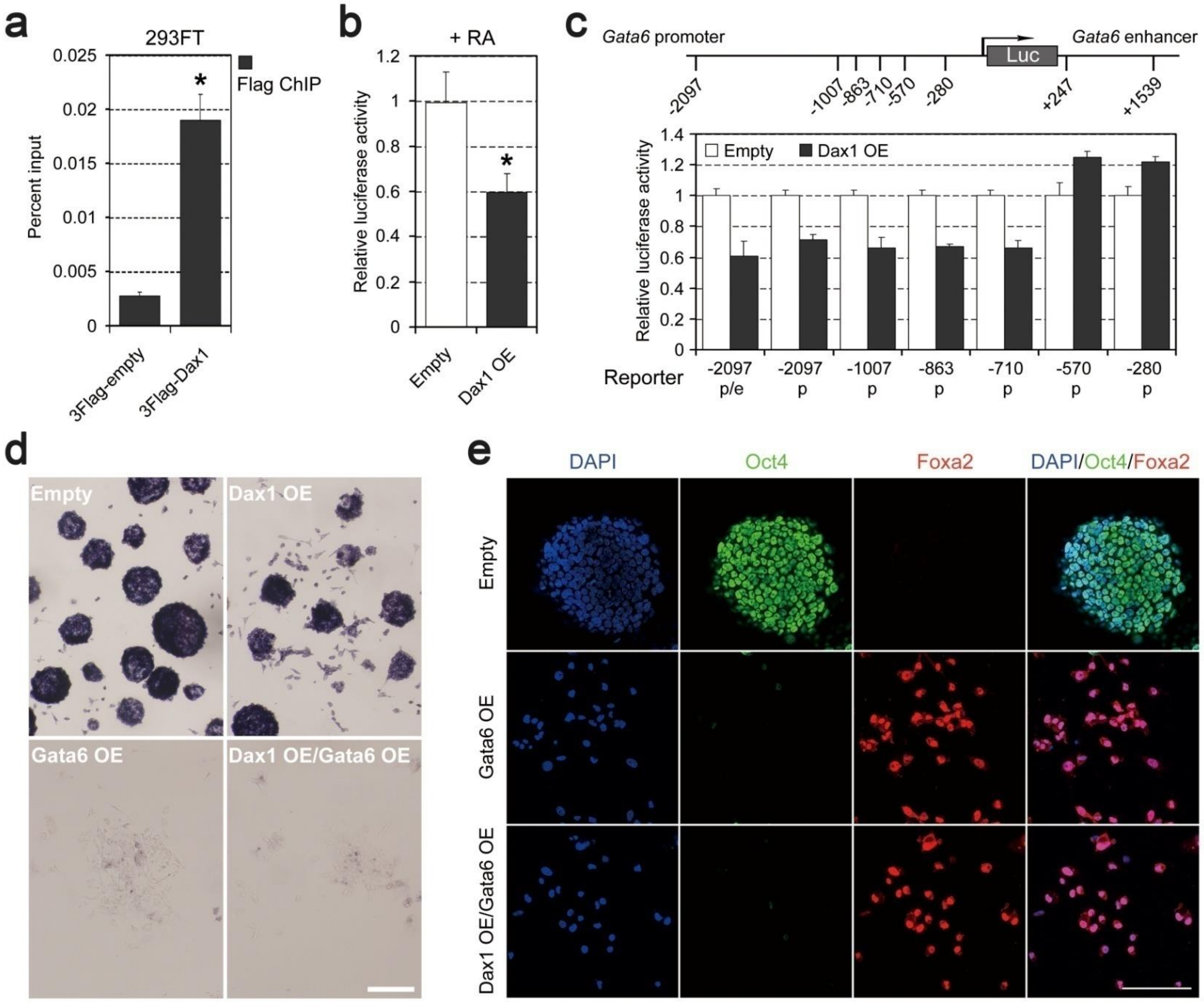

Supplementary Figure 3. The function of Dax1 in inhibiting ExEn differentiation is mediated by Gata6

(**a**) 293FT cells were cotransfected of the Gata6 promoter (positions -2097 to +13) reporter construct together with either the 3Flag-tagged Dax1 expression vector or the 3Flag-empty vector as control. ChIP was performed using anti-Flag antibody and qPCR analysis was performed with primers for the Gata6 promoter. Values are shown as percent of input DNA. Data are represented as mean ± SD; n = 3. *p <0.01.

(**b**) ESCs were induced with RA (0.1 μM) for 2 days, and then co-transfected with the Gata6 promoter (positions -2097 to +13) reporter construct and the expression vector for Dax1 (Dax1 OE) and cultured for an additional 2 days. Luciferase activity was measured and normalized against its activity with the empty expression vector (set at 1.0). Data are represented as mean ± SD; $n = 3$. *$p < 0.01$.

(**c**) Luciferase assay for ESCs transfected with the indicated Gata6 reporter constructs and empty (set at 1.0) or Dax1-overexpressing plasmids. Data are represented as mean ± SD; $n = 3$.

(**d**) AP staining of colonies formed by plating control (Empty), Dax1 OE, Gata6 OE and Dax1 OE/Gata6 OE cells at clonal density and culturing in the presence of LIF for 6 days. Scale bar, 100 μm.

(**e**) Immunofluorescence analysis of Oct4 (green) and Foxa2 (red) in the indicated cells. ESCs were transfected with each expression vector, selected for 6 days in the presence of LIF and counterstained with DAPI. Scale bar, 100 μm.

All *P*-values were calculated using Student's *t*-test.

Supplementary Figure 4

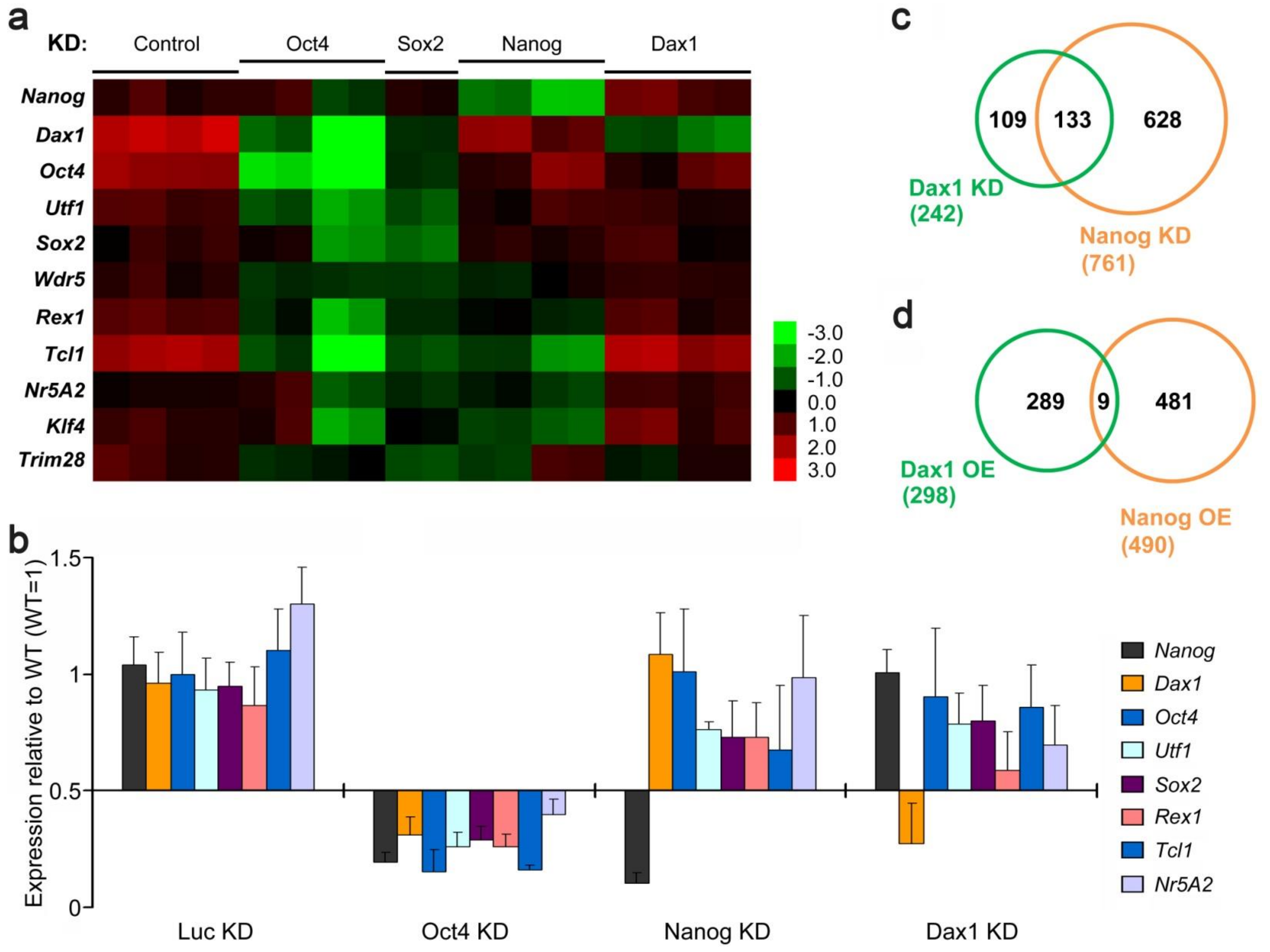

Supplementary Figure 4. Microarray analysis of gene expression associated with Dax1 and Nanog knockdown or overexpression

(**a**) Heatmap of pluripotency-associated gene expression 72 h after knockdown of Oct4, Sox2, Nanog or Dax1. Data are from the work of Ko and colleagues[1].

(**b**) qRT-PCR analysis of pluripotency-associated gene expression 72 h after knockdown of Oct4, Nanog and Dax1. All data are normalized to *Gapdh* and shown relative to WT ESCs (set at 1.0). Data are represented as mean ± SD; n = 3.

(**c**) Venn diagram of differentially expressed genes in Dax1 KD (green) and Nanog KD (orange) ESCs

(FDR <0.05 and expression fold change ≥ 1.5). Data are from the work of Ko and colleagues[1].

(**d**) Venn diagram of differentially expressed genes in Dax1 OE (green) and Nanog OE (orange) ESCs (FDR <0.05 and expression fold change ≥ 1.5). Data are from the work of Ko and colleagues[2].

False discovery rate (FDR) estimation was carried out using the Benjamini-Hochberg method.

Supplementary Figure 5

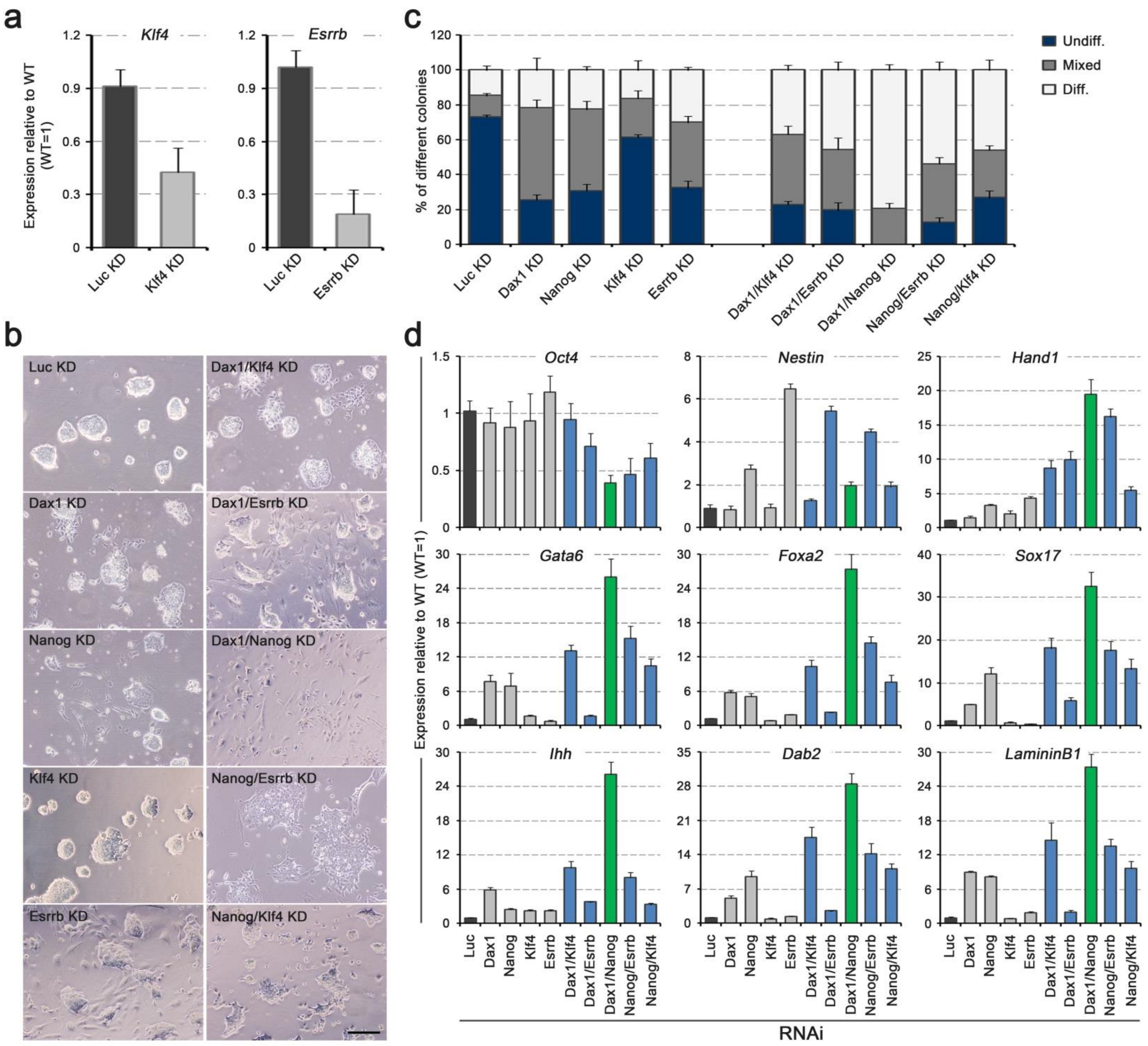

Supplementary Figure 5. Double knockdown of Dax1 and Nanog shows the strongest additive effect

(**a**) qRT-PCR analysis after Klf4 KD (left) or Esrrb KD (right). All data are normalized to *Gapdh* and shown relative to WT ESCs (set at 1.0). Data are means ± SD; n = 3.

(**b**) Representative morphologies of colonies formed by the indicated lines. Cells were cultured for 5

days with LIF. Scale bar, 100 μm.

(**c**) Quantitative analysis of colony formation assay in the indicated lines. Cells were plated at clonal density and cultured for 6 days with LIF. Colonies were fixed and stained for AP and scored as undifferentiated, mixed, or differentiated. Data are represented as mean ± SD; n = 3.

(**d**) qRT-PCR analysis of gene expression in the indicated lines after 5 days of culture with LIF. All data are normalized to *Gapdh* and shown relative to WT ESCs (set at 1.0). Data are means ± SD; n = 3.

Supplementary Figure 6

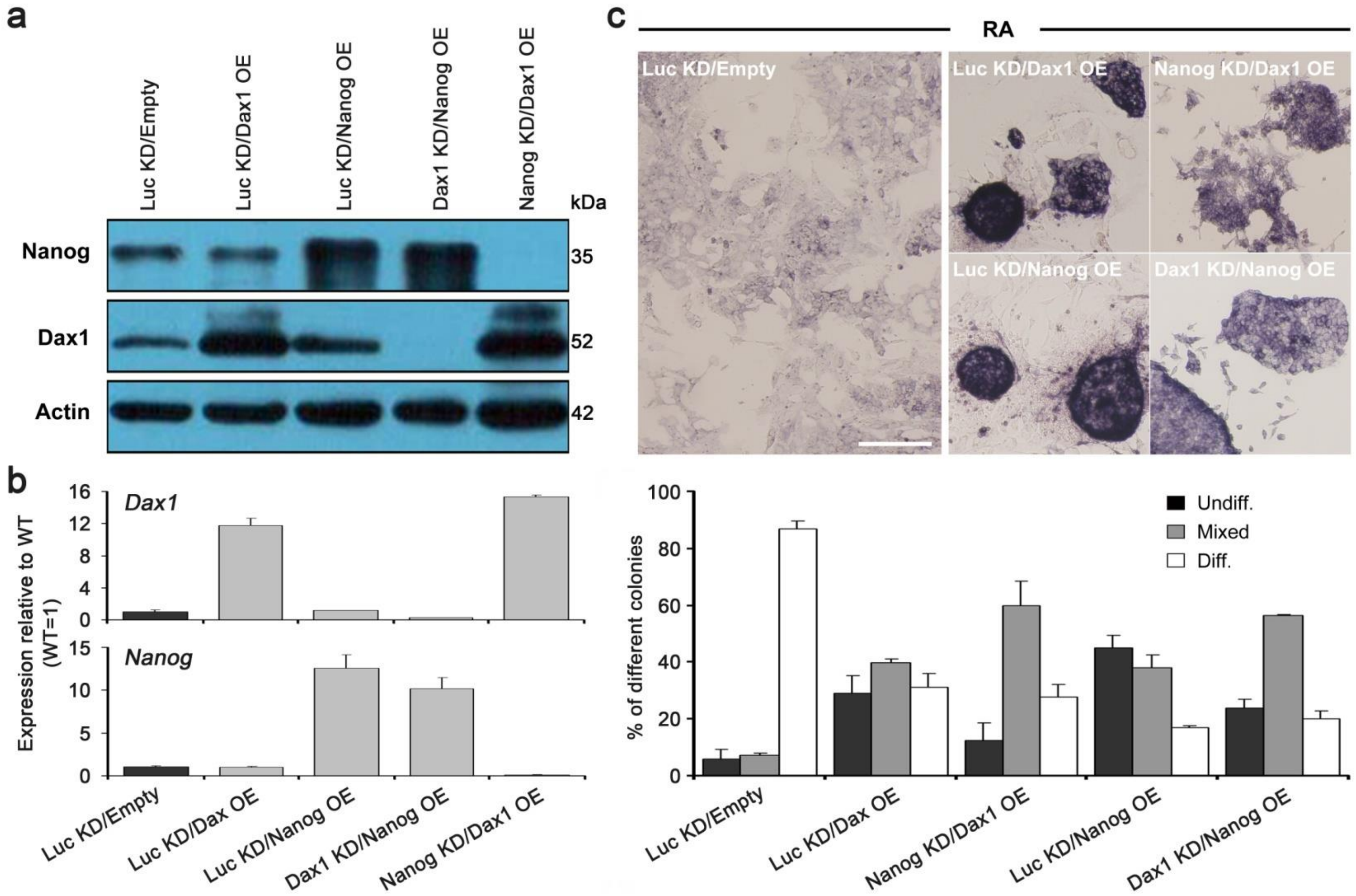

Supplementary Figure 6. Functions of Dax1 and Nanog are partially complementary

(**a**) Immunoblot analysis of Nanog and Dax1 protein in the indicated lines cultured with LIF. β-Actin was used as an internal control.

(**b**) qRT-PCR evaluating the expression of *Nanog* and *Dax1* in the indicated lines after 5 days of culture in the presence of LIF. All data are normalized to *Gapdh* and shown relative to WT ESCs (set at 1.0). Data are represented as mean ± SD; n = 3.

(**c**) AP staining of colonies formed by plating the indicated cells at clonal density and treating with RA (0.1 μM) for 4 days (upper). Percentage of colony types formed by cells is shown (lower). Data are represented as mean ± SD; n = 3. Scale bar, 100 μm.

Supplementary Figure 7

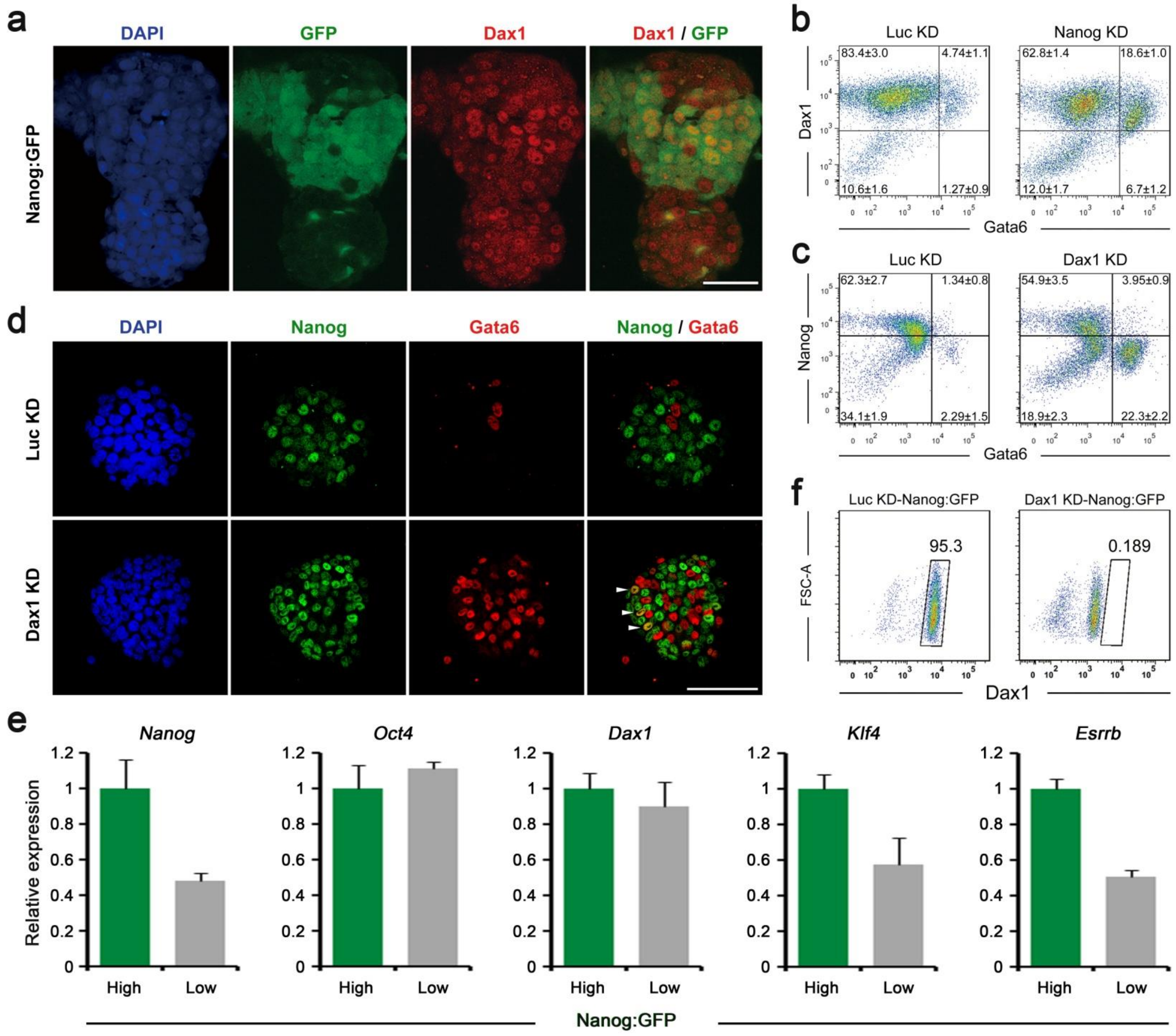

Supplementary Figure 7. Nanog heterogeneity and phenotype analysis of individual knockdown of Dax1 and Nanog in ESCs

(**a**) ESCs with GFP targeted to Nanog (Nanog:GFP cells) were stained for Dax1 (red) and counterstained with DAPI (blue). Scale bar, 100 μm.

(**b**) Dax1 and Gata6 in Luc KD or Nanog KD cells were analyzed by FACS. The shRNA transduced cells were cultured with LIF for 5 days. Numbers in quadrants indicate the percentage of each

population. Data are representative of three independent experiments.

(**c**) Nanog and Gata6 in Luc KD or Dax1 KD cells were analyzed by FACS. The shRNA transduced cells were cultured with LIF for 5 days. Numbers in quadrants indicate the percentage of each population. Data are representative of three independent experiments.

(**d**) Immunofluorescence analysis of Nanog (green) and Gata6 (red) in the indicated lines. Cells were cultured with LIF for 5 days and counterstained with DAPI (blue). Arrowheads indicate the $Nanog^{+}/Gata6^{+}$ cells. Scale bar, 100 μm.

(**e**) qRT-PCR to measure expression of *Nanog*, *Oct4*, *Dax1*, *Klf4* and *Esrrb* in the indicated FACS purified cells. All data are normalized to *Gapdh* and shown relative to the mean of $Nanog{:}GFP^{high}$ cells (set at 1.0). Data are means ± SD; n = 3.

(**f**) Flow cytometric analysis showed that the Dax1 expression was significantly silenced in the stable Dax1 KD-Nanog:GFP ESCs.

Supplementary Figure 8

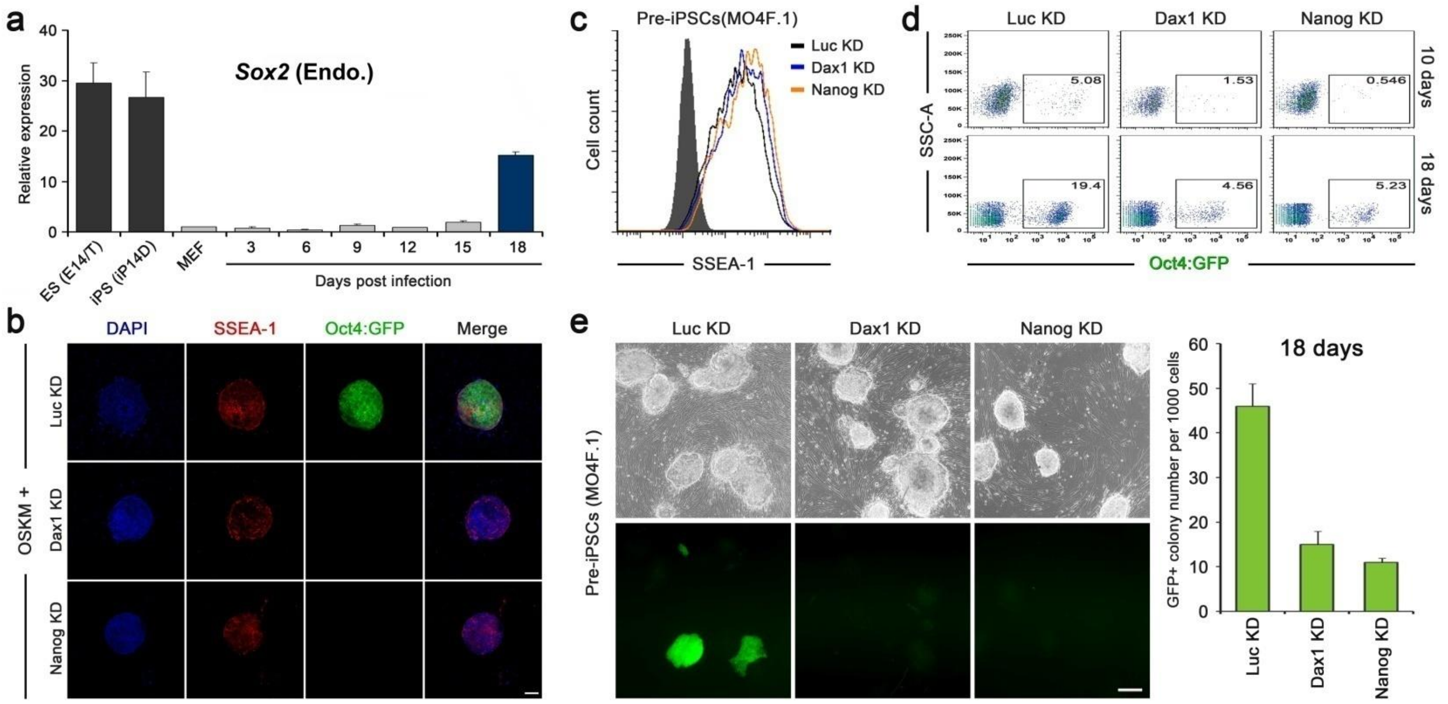

Supplementary Figure 8. Dax1 and Nanog are both required for full reprogramming to induced pluripotency

(**a**) qRT-PCR analysis of the endogenous expression levels of *Sox2* during the course of reprogramming. All data are normalized to *Gapdh* and shown relative to the mean of MEFs (set at 1.0). Data are represented as mean ± SD; n = 3.

(**b**) Immunofluorescence analysis of SSEA-1 (red) and Oct4-GFP (green) in the indicated lentiviral-infected Oct4-GFP MEF-derived cells 24 days after induction. Cells were counterstained with DAPI (blue). Scale bar, 50 μm.

(**c**) Flow cytometric analysis of SSEA-1 expression in the indicated lentiviral-infected Pre-iPSCs (MO4F.1).

(**d**) Flow cytometric analysis of Oct4-GFP reporter activity in the indicated cells. Pre-iPSCs (MO4F.1) were infected with the respective lentiviruses and cultured in serum/LIF for 10 or 18 days. Numbers indicate the percentage of Oct4-GFP^{+} cells.

(**e**) Phase and Oct4-GFP images of the indicated lentiviral-infected Pre-iPSCs (MO4F.1). Cells were cultured for 18 days in serum/LIF. Scale bar, 100 μm. Numbers of Oct4-GFP$^+$ colonies are shown in the bar graph next to the images. Data are represented as mean ± SD; n = 3.

Supplementary Figure 9

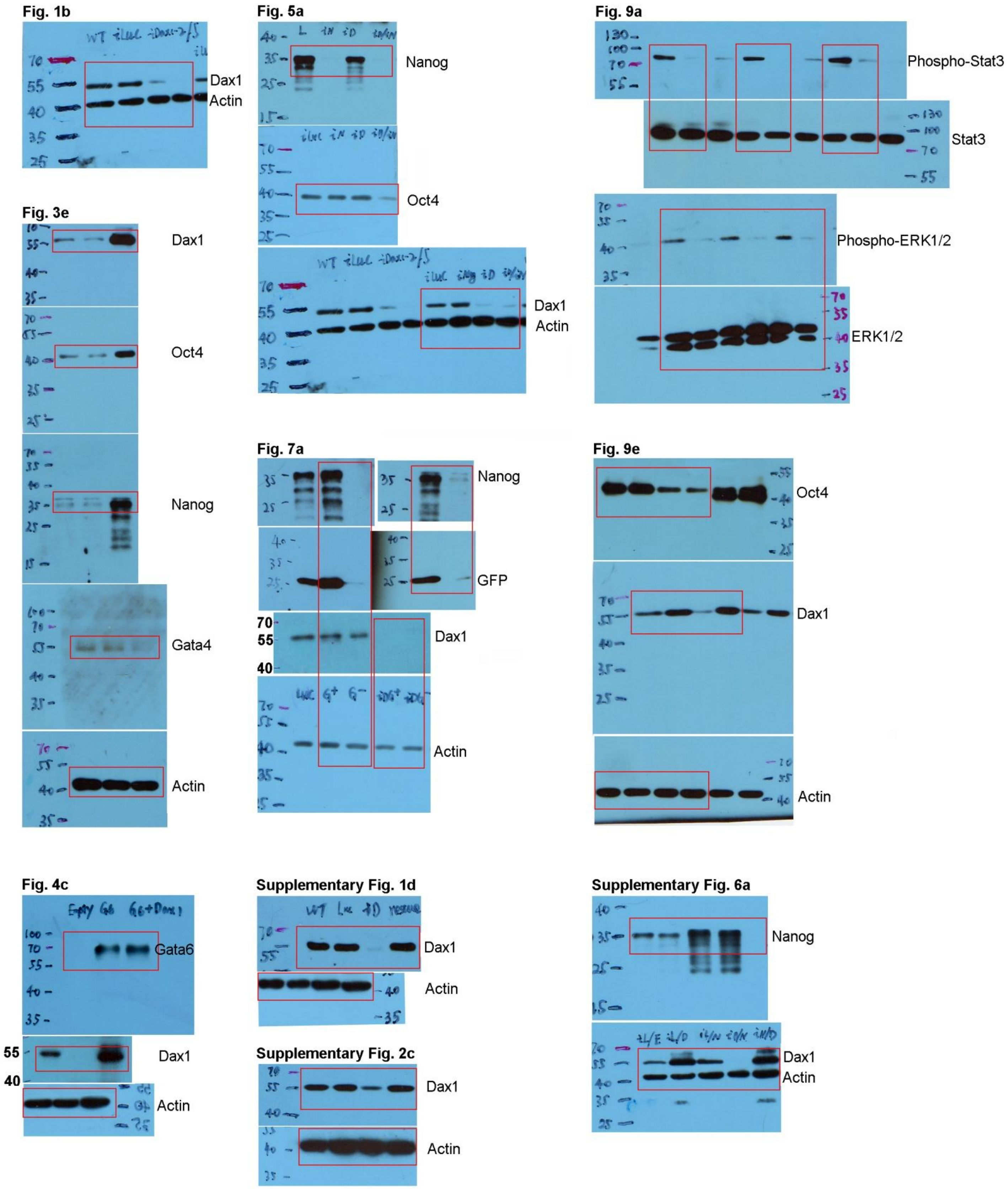

Supplementary Figure 9. Scans of the immunoblots. Boxes highlight lanes used in figures.

Supplementary Tables

Supplementary Table 1. Summary of previous work on Dax1 in ESCs

Study	Methods for Functional Analysis	Time of Analysis	Phenotype
(Niakan et al., 2006)[3]	conditional KO & KD, siRNA transfection	2 days	ExEn differentiation
(Wang et al., 2006)[4]	KD, retroviral shRNA	4 days	multilineage differentiation
(Khalfallah et al., 2009)[5]	KD, siRNA transfection	4 days	multilineage differentiation
(Martello et al., 2012)[6]	KD, siRNA transfection	2 days	no loss of pluripotency
(Sun et al., 2009)[7]	OE, episomal supertransfection	8 days	TrE differentiation
(Kelly et al., 2010)[8]	OE, conventional transfection	2 days	no loss of pluripotency

KO, knockout; KD, knockdown; OE, overexpression; ExEn, extra-embryonic endoderm; TrE, trophectoderm;

Supplementary Table 2. Cell lines used in the study

Cell Line	Type	Reference	Genetic Modification
R1	mESC	(Nagy et al., 1993)[9]	
CCE	mESC	(Keller et al., 1993)[10]	
		(Robertson et al., 1986)[11]	
E14/T	mESC	(Aubert et al., 2002)[12]	
Nanog:GFP	mESC	(Hatano et al., 2005)[13]	A *GFP-IRES-Puro-pA* cassette has been introduced into one *Nanog* allele by homologous recombination
iP14D	iPSC	(Zhao et al., 2009)[14]	Express a randomly integrated transgene in which GFP is controlled by Oct4 regulatory elements

Supplementary Table 3. Primers for qRT-PCR analysis

Gene	Primer Sequence (5' - 3')	
	Forward	Reverse
GAPDH	AGGTCGGTGTGAACGGATTTG	TGTAGACCATGTAGTTGAGGTCA
Oct4	CACGAGTGGAAAGCAACTCA	AGATGGTGGTCTGGCTGAAC
Nanog	TCTTCCTGGTCCCCACAGTTT	GCAAGAATAGTTCTCGGGATGAA
Sox2	AGGCAAACGTTCTAGATTGTAC	ACCAACGATATCAACCTGCATG
Rex1	CCCCAAATACCACTGACCAA	AACTCACCTCGTATGATGCA
Dppa3	CTGAAAGACCCTATAGCAAAGA	CTCACTGTCCCGTTCAAACTCA
Dppa4	CTGGGTTGTAGTGTGTTGTTAG	GCCACAAGCCAGATTTAGTGTA
Sall4	GGCAATCCTACAATCACGAGAG	CCATGAGGTCACACAAAGCTTC
Eras	CAAGCAAGAAGACCCGACACCA	GATGTCTGTGGTAACTTGGTC
Utf1	TTGAATACCGCGTTGCTGCAGA	AGAAGAGGACTGATAACAAAGC
Nr5A2	GAGCTCTTGATTCTCGATCACA	AACTCCCGCTGATCGAACTGAA
Tcl1	TTTATCACGGACTGGCATTG	GGGTCTGGGTTATTCATCGT
Fgf5	ACGAGGAGTTTTCAGCAACAA	CGCGGACGCATAGGTATTAT
Sox1	TGAACGCCTTCATGGTGTGGTC	GCGCGGCCGGTACTTGTAAT
Nestin	AGATCGCTCAGATCCTGGAA	AGGTGTCTGCAAGCGAGAGT
Brachyury[5]	CTGCGCTTCAAGGAGCTAAC	CCAGGCCTGACACATTTACC
Flk1	TTTGGCAAATACAACCCTTCAGA	GCAGAAGATACTGTCACCACC
Goosecoid	ATGCTGCCCTACATGAACGT	CAGTCCTGGGCCTGTACATT
Sox17	GTGGACCGCACGGAATTCGAA	GCAATAGTAGACCGCTGAGCTA
Foxa2	GTGTACTCCAGGCCTATTATGA	GGTGGCTACTTTTCCTCAAAG
Gata6	AAAGCTTGCTCCGGTAACAG	TTCTCCCACTGCAGACATCA
Afp	GGCGATGGGTGTTTAGAAAG	CAGCAGCCTGAGAGTCCATA
Ihh	TTCTTCACACGCATTCCATCTG	AGCCTCTCTTCATACTGTGATC
LamininB1	GAAAGGAAGACCCGAAGAAAAGA	CCATAGGGCTAGGACACCAAA
Dab2	GGATAAAGAGGTCAAGGAAGTG	TGGTCTACATGCTCCTGAGGAA
Cdx2	CAAGGACGTGAGCATGTATCC	GTAACCACCGTAGTCCGGGTA
Eomes	GGTTGGACCTTGAGTTAACAG	AGCTAGCCACTTAAACATCAG
Hand1	TGAACTCAAAAAGACGGATGG	CTTTAATCCTCTTCTCGCCG
Dax1	GCAGGGCAGCATCTTATACAGC	GCTCTTCACCGCACACATAG
Klf4	CTAAATGATGGTGCTTGGTGA	TGGCTTAGGTCATCAATGTAG
Esrrb	ATCTCTTTCTCTCGTTCCCTAA	CGTATTTCTCAAGCTCTGTCCA

Supplementary Table 4. Antibody details

	Antibody	Source	Cat. no.	Dilution
**Primary**	Dax1	active motif	39983	1:100 (IF, FCM); 1:1000 (WB)
	Oct4	Santa Cruz	sc-5279	1:100 (IF, FCM); 1:1000 (WB)
	Nanog	Abcam	ab80892	1:100 (IF, FCM); 1:1000 (WB)
	Gata6	RD system	AF1700	1:100 (IF, FCM); 1:1000 (WB)
	Gata4	Santa Cruz	sc-1237	1:100 (IF, FCM); 1:1000 (WB)
	Foxa2	Santa Cruz	sc-9187	1:100 (IF)
	Cdx2	BioGenex	MU392A-UC	1:100 (IF)
	Nestin	Beyotime	AN203	1:100 (IF)
	SSEA-1	eBioscience	14-8813-80	1:100 (IF, FCM)
	STAT3	eBioscience	14-6013-81	1:1000 (WB)
	Phospho-STAT3	cell signaling	#9131	1:1000 (WB)
	p44/42 MAPK (ERK)	Beyotime	AM076	1:1000 (WB)
	Phospho-p44/42 MAPK (ERK)	Beyotime	AM071	1:1000 (WB)
	β-Actin	Sigma	A1978	1:2000 (WB)
	FLAG	Beyotime	AF519	1:200 (ChIP); 1:1000 (WB)
**Secondary**	Donkey anti-Rabbit IgG (H&L), DyLight®650 Conjugate	ImmunoReagents	DkxRb-003-E650NHSX	1:200 (IF, FCM)
	Donkey anti-Goat IgG (H&L), DyLight®594 Conjugate		DkxGt-003-E594NHSX	
	Donkey anti-Mouse IgG (H&L), DyLight®488 Conjugate		DkxMu-003-D488NHSX	
	Goat anti-Mouse IgG/HRP	zhongshan	ZDR-5307	1:4000 (WB)
	Goat anti-Rabbit IgG/HRP		ZDR-5306	
	Rabbit anti-Goat IgG/HRP		ZDR-5308	

Supplementary Table 5. Target sequences of shRNA against target genes

Name	Target Gene	Sequence (sense strand)	Starting Nucleotide from ATG (bp)
Dax1 KD-2	*Nr0B1* (CDS)	GCAGCGTCTGATCACACTCAA	732
Dax1 KD-5	*Nr0B1* (3' UTR)	GAAGAAATACTTTAGTTACAA	1552
Nanog KD	*Nanog*	GAACTATTCTTGCTTACAA[15]	99
Oct4 KD	*Oct4/Pou5f1*	GAAGGATGTGGTTCGAGTA[15]	396
Klf4 KD	*Klf4*	GAGGAACTCTCTCACATGA[16]	109
Esrrb KD	*Esrrb*	GATTCGATGTACATTGAGA[17]	1120
Luc KD	*Luciferase*	GACGAACACTTCTTCATCG	1285

Supplementary Table 6. **Primers for plasmid construction**[a]

Name	Primer Sequence (5' - 3')	
	Forward	Reverse
pPyCAGIP-mDax1	GAATTCGGATCCACCATGGCGGGTGAGGACCACC	GGATCCGAATTCTCACAGCTTTGCACAGAGCA
pCAG-2AH-mNanog	GGTACCATGAGTGTGGGTCTTC	GTTAACTATTTCACCTGGTGGA
pCAG-2AH-mOct4	AGATCTGGTACCATGGCTGGACACCTG	ACGCGTGTTAACGTTTGAATGCATGGGA
pCAG-2AH-mGata6	CGGGGTACCAAAAGTCAGGTTGGAGTAG	GGCCAGGGCCAGAGCACAC
pGL3-Gata6P2097[b]	CCCATCTAGAACCTTGAAGGTCGTTCACAGCCAG	ACCGAGTCGACGTCGGAACTGCTCTCTACAAG
pGL3-Gata6P1007[c]	CCCATCTAGACCGTATCAGTTTGTACACAG	
pGL3-Gata6P863[d]	CCCATCTAGACCTGAAGTCCAGAGAAAGCTC	
pGL3-Gata6P710[e]	CCCATCTAGAACTCTACAAAACCTCCCAG	
pGL3-Gata6P570[f]	CCCATCTAGAGTTTAAGGTCAGTTACTAGCAA	
pGL3-Gata6P280[g]	CCCATCTAGAGTTACAGCGCTGGATGATTATG	
pGL3-Gata6E[h]	CCGAAGATCTTCGGTTGTTTCCTCCTTTGTTTGC	ACGCAGTCGACCCTAAGGTGGGAGTGAGGAACAAG

[a]see Extended Experimental Procedures for construction details.
[b-g]pGL3 vectors containing serial deletion fragments of the Gata6 promoter.
[h]the pGL3 vector containing the Gata6 enhancer.

Supplementary Table 7. Primers for ChIP-qPCR analysis

Name	Primer Sequence (5' - 3')	
	Forward	Reverse
pGata6-1	TGCCACCTCAATGACCTTTCCG	GGTTCTCTTTCTGCCTGGCCTC
pGata6-2	CCTTGAAGGTCGTTCACAGCCA	GCAATTCATTAAAGCTTCGGACA
pGata6-3	GTTACAGCGCTGGATGATTATG	AGCTGCTAGCCCTGTCCTCAGA
pGata6-4	GCAGAAGCGGGATATTTAAGGA	CCTAAGGTGGGAGTGAGGAACA
pGata6-5	GGCTGTCACCCTTTCTACCACTG	GCACAAGCTATCTTCTCAGTCA
pGata6-6	TAGGTGCTCAGGGTTGGTGTG	GGGAAGTGGCAGTTAGTTGGA
pDax1-1	CGATCGAAGAATGGATACAGTA	GGTTCCTCCTTCCTTCCACAA
pDax1-2	CCTGATTATCTGCTTACTGGAA	TGCATACAACCTTCAACTTCAA
pDax1-3	CTAGGTGGGTTAAGAAGAAAGA	AGGACAAGGGATCAATTGAGGA
pDax1-4	AGAGGTCAGAGTCTAAGTTAATG	TGTGTGGCCATGACCTCGAAAG
pDax1-5	CAGACAGACGAACAGACAGGAA	GGACTGTCCAGTCATTAGATTG
pDax1-6	GCTATTCAGGTCCATTCACTTA	ACAGTGGAATGGACCTATTTCA
pDax1-7	AGCAGGCCCTTCAACGAACAGA	CCAGAAATGAGAGTGGTTAGGTA
pOct4-1	CCTATTCCTACCTTCAGCTTCTA	CCTTGTCTTGGTTTTCTTGCCTA
pOct4-2	CATACTTGAACTGTGGTGGAGA	GCAGATTAAGGAAGGGCTAGGA
pOct4-3	CAGATATTTCTTCTCTCTACCCA	CCAGGTTGGTAGATTAGACACTA
pOct4-4	GCCATCTTAAGATCATTCTGTG	AGCTTAACTAAGGTTCAAATCTC
pOct4-5	TCTGTGTCCTTATTCTGCTGCTA	TGCTAAGGGAGCATTGACTGGA
pOct4-6	CAAGGAACTGGATTTATAGAGA	TTGGAAGGCAGGGATGGATAGA

Supplementary references

1. Nishiyama, A. *et al.* Systematic repression of transcription factors reveals limited patterns of gene expression changes in ES cells. *Sci Rep* **3**, 1390 (2013).
2. Nishiyama, A. *et al.* Uncovering early response of gene regulatory networks in ESCs by systematic induction of transcription factors. *Cell Stem Cell* **5**, 420-33 (2009).
3. Niakan, K.K. *et al.* Novel role for the orphan nuclear receptor Dax1 in embryogenesis, different from steroidogenesis. *Mol Genet Metab* **88**, 261-71 (2006).
4. Wang, J. *et al.* A protein interaction network for pluripotency of embryonic stem cells. *Nature* **444**, 364-8 (2006).
5. Khalfallah, O., Rouleau, M., Barbry, P., Bardoni, B. & Lalli, E. Dax-1 knockdown in mouse embryonic stem cells induces loss of pluripotency and multilineage differentiation. *Stem Cells* **27**, 1529-37 (2009).
6. Martello, G. *et al.* Esrrb is a pivotal target of the Gsk3/Tcf3 axis regulating embryonic stem cell self-renewal. *Cell Stem Cell* **11**, 491-504 (2012).
7. Sun, C. *et al.* Dax1 binds to Oct3/4 and inhibits its transcriptional activity in embryonic stem cells. *Mol Cell Biol* **29**, 4574-83 (2009).
8. Kelly, V.R., Xu, B., Kuick, R., Koenig, R.J. & Hammer, G.D. Dax1 up-regulates Oct4 expression in mouse embryonic stem cells via LRH-1 and SRA. *Mol Endocrinol* **24**, 2281-91 (2010).
9. Nagy, A., Rossant, J., Nagy, R., Abramow-Newerly, W. & Roder, J.C. Derivation of completely cell culture-derived mice from early-passage embryonic stem cells. *Proc Natl Acad Sci U S A* **90**, 8424-8 (1993).

10. Keller, G., Kennedy, M., Papayannopoulou, T. & Wiles, M.V. Hematopoietic commitment during embryonic stem cell differentiation in culture. *Mol Cell Biol* **13**, 473-86 (1993).

11. Robertson, E., Bradley, A., Kuehn, M. & Evans, M. Germ-line transmission of genes introduced into cultured pluripotential cells by retroviral vector. *Nature* **323**, 445-8 (1986).

12. Aubert, J., Dunstan, H., Chambers, I. & Smith, A. Functional gene screening in embryonic stem cells implicates Wnt antagonism in neural differentiation. *Nat Biotechnol* **20**, 1240-5 (2002).

13. Hatano, S.Y. *et al.* Pluripotential competence of cells associated with Nanog activity. *Mech Dev* **122**, 67-79 (2005).

14. Zhao, X.Y. *et al.* iPS cells produce viable mice through tetraploid complementation. *Nature* **461**, 86-90 (2009).

15. Ang, Y.S. *et al.* Wdr5 mediates self-renewal and reprogramming via the embryonic stem cell core transcriptional network. *Cell* **145**, 183-97 (2011).

16. Jiang, J. *et al.* A core Klf circuitry regulates self-renewal of embryonic stem cells. *Nat Cell Biol* **10**, 353-60 (2008).

17. Loh, Y.H. *et al.* The Oct4 and Nanog transcription network regulates pluripotency in mouse embryonic stem cells. *Nat Genet* **38**, 431-40 (2006).
